# Teamwork of clustered low-affinity κB sites and accessory factors regulates transcriptional strength of NF-κB RelA dimers

**DOI:** 10.1093/nar/gkaf846

**Published:** 2025-10-08

**Authors:** Shandy Shahabi, Tapan Biswas, Yuting Shen, Rose Sanahmadi, Yaya Zou, Gourisankar Ghosh

**Affiliations:** Department of Chemistry and Biochemistry, University of California San Diego, 9500 Gilman Drive, La Jolla,CA 92093, United States; Department of Chemistry and Biochemistry, University of California San Diego, 9500 Gilman Drive, La Jolla,CA 92093, United States; Department of Chemistry and Biochemistry, University of California San Diego, 9500 Gilman Drive, La Jolla,CA 92093, United States; Department of Chemistry and Biochemistry, University of California San Diego, 9500 Gilman Drive, La Jolla,CA 92093, United States; Department of Chemistry and Biochemistry, University of California San Diego, 9500 Gilman Drive, La Jolla,CA 92093, United States; Department of Chemistry and Biochemistry, University of California San Diego, 9500 Gilman Drive, La Jolla,CA 92093, United States

## Abstract

Non-consensus binding sites of transcription factors (TFs) are often observed within the regulatory elements of genes; however, their effect on transcriptional strength is unclear. Within the promoters and enhancers of NF-κB-responsive genes, we identified clusters of non-consensus κB DNA sites, many exhibiting low affinity for NF-κB *in vitro*. Deletion of these sites demonstrated their collective critical role in transcription. We explored how these “weak” κB sites exert their influence, especially given the typically low nuclear concentrations of NF-κB. Using proteomics approaches, we identified additional nuclear factors, including other DNA-binding TFs, that could interact with κB site-bound NF-κB RelA. ChIP-seq and RNA-seq analyses suggest that these accessory TFs, referred to as the TF-cofactors of NF-κB, facilitate dynamic recruitment of NF-κB to the clustered weak κB sites. Overall, the occupancy of NF-κB at promoters and enhancers appears to be defined by a collective contribution from all κB sites, both weak and strong, in association with specific cofactors. This congregation of multiple factors within dynamic transcriptional complexes is likely a common feature of transcriptional programs.

## Graphical Abstract

**Graphical Abstract:**
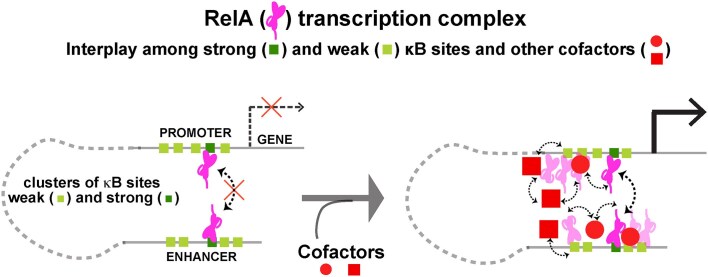

## Introduction

Transcription is triggered by transcription factors (TFs) binding to specific short DNA response elements (REs) located commonly within the non-coding promoter and enhancer regions [1, 2]. Our understanding of interactions between eukaryotic TFs and their cognate REs in genes has improved significantly due to genome-wide and imaging analyses of the last two decades [3–5]. These studies have also underscored various unresolved aspects about sequence-specific DNA recognition by TFs in cells [5–7]. The first intriguing aspect is the presence of multiple TF-binding sites of a wide range of affinities in a promoter, which raises the question of the possible roles of weak binding sites in transcription. The second intriguing aspect is the association of many TFs to promoter and enhancer regions that lack cognate DNA binding sites of these TFs, hinting at their non-DNA binding roles in gene expression [8, 9]. It is unclear whether these auxiliary TFs regulate recruitment of primary TF to the weak DNA REs. We addressed these questions through investigation of transcriptional programs mediated by NF-κB RelA, a suitable model system due to availability of information from decades of rigorous studies.

Among the NF-κB family of dimeric TFs, the p50:RelA heterodimer and the RelA:RelA homodimer are most ubiquitous and well-studied [10, 11]. These dimers bind with high affinity to ∼10-bp sequences (κB sites), with a general consensus of GGGRNNYYCC, defined over 35 years ago [12, 13]. Several studies indicate binding of various NF-κB dimers to thousands of degenerate κB sites with a wide gradient in affinity [10, 14–16]. Some of the weak sites are not easily recognizable nonetheless bearing specificity to specific NF-κB dimers. The promoters of known NF-κB target genes often contained several of these weak binding sites; however, the functional importance of the vast majority of these weak sites remains unexplored. The promoters of prominent NF-κB regulated genes e.g. E-selectin, Cxcl10, Ccl2, IL-1β, TNF-α, and Nfkbia, all contain multiple κB sites, including many of lower affinity. Cell-based reporter assays indicated the necessity of some of these weak sites in optimal reporter expression [10, 17]. However, the prevalence of bona fide weak κB sites in native promoters and enhancers of the earlier-mentioned and other NF-κB-regulated genes is largely unclear, and the lower limit of binding affinity of a weak κB site for it to be functional is not defined. Essential roles of weak DNA binding sites of various TFs within the enhancer regions have been reported during development [18–22], where suboptimal binding of TFs to the weak sites appears to provide specificity for gene expression. For the TF ETS, a single nucleotide change in a binding site causing only a small enhancement in the binding affinity is observed to cause pathogenicity [22]. However, the mechanistic possibilities of how multiple binding sites within a promoter/enhancer can act in concert, either for the strength or the specificity of transcription, remain largely unexplored.

TFs have also been observed to require auxiliary factors for cognate DNA recognition [23]. Previous studies revealed that NF-κB is recruited along with additional factors [10, 24–26]. The associated factors during RelA recruitment are promoter and cell-type specific, although it is unclear whether they play a role in facilitating occupancy [24, 25, 27].

We set out to clarify these unresolved issues in transcription through investigating the role of weak κB sites and supporting factors in the recruitment of NF-κB RelA to DNA and consequently transcription. Through analysis of ChIP-seq data of NF-κB p50:RelA and RelA:RelA dimers during stimulus-dependent expression of NF-κB-responsive genes, we observed that multiple κB sites are present in clusters within ∼150-bp-long DNA segments of promoter and enhancer regions. Additionally, we observed many of these κB sites to be weak i.e. displaying a barely detectable affinity *in vitro*. However, these sites appear to play essential roles in recruiting NF-κB to target genes and driving transcription. Additionally, we observe that numerous protein factors, including other TFs (TF-cofactors), RNA-binding proteins, and enzymes, associate with NF-κB RelA on DNA. Further investigation of these TF-cofactors revealed their critical role in enhancer- and promoter-specific recruitment of RelA.

## Materials and methods

### Antibodies and reagents

The RelA (8242), H3 (9715), Lamin B1 (D4Q4Z), and Gapdh (14C10) antibodies for western blot and electrophoretic mobility shift assay (EMSA) supershift assays were purchased from Cell Signaling Technology. The Nfat5 (PA1-023), Cux1 (PA5-36355), and Smad4 (MA5-15682) antibodies were purchased from Thermo Fisher Scientific. The Tead3 (A7454) antibody was purchased from ABclonal. The IκBα (0040) and tubulin (0119) antibodies were purchased from Bio Bharati Life Science. The FLAG (F1804) antibody was purchased from Sigma–Aldrich. The Nfatc1 (7294) antibody was purchased from Santa Cruz Biotechnology. The RelA antibody (C15310256) used for ChIP-Seq was purchased from Diagenode. Mouse TNF-α was obtained from Bio Bharati Life Science and used at a final concentration of 10 ng/mL for the indicated timepoints for stimulation.

### Mammalian cell culture and generation of stable cell lines

Wild-type (WT) and genetically modified mouse embryonic fibroblast (MEF), HeLa S3, and HEK 293T cell lines were cultured in Dulbecco’s modified Eagle’s medium (DMEM; Corning) supplemented with 10% fetal bovine serum (Corning), 1% penicillin streptomycin glutamine (Gibco), and appropriate antibiotics.

For generation of stable CRISPR-modified MEF cell lines, gRNA targeting a 20 nt genomic region adjacent to an NGG (*N* = any nucleotide) Cas9 protospacer adjacent motif was first annealed and cloned into BsmbI (NEB) digested pLentiCRISPRv2 vector. Sanger sequencing was performed to verify cloning. The different pLentiCRISPRv2 gRNA constructs were then cotransfected with pMDLg/pRRE, pCMV-VSV-G, and pRSV-Rev expression constructs into HEK293T cells using TransIT-Lenti transfection reagent (Mirus) for generation of lentiviral particles. After 24 h, supernatant containing virus was collected and filtered through a 0.4 μm filter, and this was used to infect MEF cells at a dilution of 1:10 in the presence of 10 ng/μL polybrene (MilliporeSigma) for 48 h. MEF cells were then selected by growing in media containing 5 μg/mL puromycin. Stable shRNA knockdown (KD) MEF cells were generated similarly, except for the annealed oligos for targeting shRNA were cloned within AgeI and EcoRI sites of pLKO.1 TRC vector, which were then cotransfected with pMDLg/pRRE, pCMV-VSV-G, and pRSV-Rev expression constructs into HEK293T cells for generation of viral particles.

### Protein expression and purification

Full-length His-RelA (1-551) was expressed in *Spodoptera frugiperda* 9 (Sf9) insect cells using the Bac-to-Bac baculoviral expression system as previously described [12]. Briefly, baculovirus-infected Sf9 cells were sonicated in lysis buffer containing 25 mM Tris–HCl, pH 7.5, 500 mM NaCl, 5% glycerol, 0.1% NP-40, 20 mM imidazole, 5 mM β-mercaptoethanol, and 0.5 mM PMSF. The lysate was clarified by centrifugation at 40 000 × *g* for 30 min and incubated with Ni-NTA agarose beads (Thermo Fisher Scientific). Beads were extensively washed with lysis buffer without PMSF, and RelA was eluted with lysis buffer containing 250 mM imidazole and no PMSF. Appropriate elution fractions were pooled, concentrated, and applied to a HiLoad 16/60 Superdex 200 (Cytiva) size-exclusion column equilibrated with buffer containing 25 mM Tris–HCl, pH 7.5, 200 mM NaCl, 5% glycerol, and 1 mM DTT. Peak fractions were pooled, concentrated, and aliquots were flash frozen in liquid nitrogen.

For preparation of recombinant p50:RelA heterodimer, His-tagged p50 (1-435) was transformed into *Escherichia coli* Rosetta (DE3) cells. Three to five colonies were used to inoculate a 5 mL LB starter culture overnight at 37°C, which was then used to inoculate 2 L of LB the next day. Cells were grown at 37°C to an attenuance (600 nm) of 0.2 and induced with 0.25 mM isopropyl β-d-thiogalactpyranoside (IPTG) for 18 h at 25°C. Cells were harvested by centrifugation at 3000 × *g* for 10 min and sonicated in lysis buffer containing 25 mM Tris–HCl, pH 7.5, 250 mM NaCl, 10% glycerol, 0.1% NP-40, 10 mM imidazole, 0.25 mM PMSF, and 5 mM β-mercaptoethanol. Lysate was clarified by centrifugation at 40 000 × *g* for 30 min and incubated in batch with Ni-NTA agarose beads (Thermo Fisher), and beads were washed extensively with lysis buffer containing 20 mM imidazole and without PMSF prior to elution of recombinant His-tagged p50 in lysis buffer containing 250 mM imidazole without PMSF. The eluted p50 was concentrated and mixed with His-tagged RelA (1-551) in a 1:1 molar ratio at a final concentration of 0.1 mg/mL in buffer containing 25 mM Tris–HCl, pH 7.5, 250 mM NaCl, 10% glycerol, and 1 mM DTT, and incubated for 1 h at room temperature. The p50:RelA heterodimer formed was then concentrated and separated on a HiLoad 16/60 Superdex 200 size-exclusion column equilibrated in buffer containing 25 mM Tris–HCl, pH 7.5, 200 mM NaCl, 5% glycerol, and 1 mM DTT. Peak fractions of pure heterodimer were collected, concentrated, and aliquots were flash frozen in liquid nitrogen.

### Crystallization of RelA:κB complexes and structure determination

For crystallization of RelA:κB-DNA complexes, κB-DNA duplexes were generated as previously described [13]. Complexes were formed by incubating untagged RelA(19–304) at a final concentration of ∼150 micromolar with κB-DNA duplex in 1:1.2 molar ratio in buffer containing 25 mM Tris–HCl, pH 7.5, 50 mM NaCl, and 1 mM DTT for 15 min at room temperature. Crystals were obtained by hanging drop vapor diffusion method at 18°C—mixing 1 μl of complex with 1 μl of reservoir solution containing MES, pH 5.5, 2 mM calcium chloride, 50 mM ammonium chloride, 14% PEG3350, 1 mM spermine, and 0.05% octyl β-d-glucopyranoside. Crystals were nucleated around the 4th day and reached a maximum size of around 50 × 50 × 200 μm around the 10th day. Crystals were soaked for 5 min in the mother liquor without DTT containing 21% ethylene glycol for cryo-protection and flash frozen in liquid nitrogen for subsequent diffraction data collection at the synchrotron source. Structure was determined by molecular replacement technique using CCP4i [28].

### Analysis of RelA binding sites in promoter from ChIP-seq data

We analyzed previously reported RelA ChIP-Seq data sets of TNF-α stimulated MEF cells [23] to correlate genomic RelA DNA binding strength (ChIP-Seq peak intensity) with calculated motif strength (RelA motif enrichment) from established RelA-dependent target genes. The JASPAR TF binding database was used for initial RelA motif identification within RelA ChIP-Seq peaks (PMC10767809). The strength of RelA binding was calculated by quantifying peak intensity at strongest RelA motif within ChIP-Seq peaks, and the strongest motif associated with a gene (defined by proximity to TSS) was considered to sort the genes. The genomic regions centered around ChIP-Seq peaks (±500 bp) were analyzed for the presence of other RelA motifs of various affinities. The affinities of RelA sequence motifs defined using *Z*-scores were previously estimated in a SELEX study with p50:RelA [12]. A *Z*-score boundary of 6 (equivalent to approximate *in vitro* binding affinity of greater than 300 nM) was used to separate strong versus weak RelA-binding κB sites. Cumulative *Z*-score was calculated with a dynamic window ranging from 10 to 500 bp surrounding the strongest RelA motif for an individual RelA ChIP-seq peak, and a Pearson correlation analysis between cumulative *Z*-score and length of analysis window was performed.

### RNA-seq analyses

RNA-seq experiments were performed in triplicate. In brief, five million control or KD MEF cells were plated in a single well of six-well plates (GenClone). After overnight incubation, the cells were stimulated by adding TNF-α (BioBharati) to medium at a concentration of 10 ng/mL for 1 h. Cells were washed with PBS on well and treated with TRIzol (Invitrogen) to isolate RNA following the manufacturer’s recommendation. Isolated RNA was quantified using ND-1000 spectrophotometer (Nanodrop), its integrity was assessed with Tapestation (Agilent), and DNA libraries were prepared using 1 μg of total RNA using KAPA mRNA Hyperprep Kit (Roche) with KAPA Unique Dual-Indexed adapters (Roche). DNA libraries were PCR amplified (8 cycles) prior to analysis by Tapestation (Agilent). Next, libraries were quantified by dsDNA Qubit (Thermo Fisher Scientific), pooled, and put through 100 bases of paired-end sequencing using the NovaSeq 6000 S4 sequencer (Illumina). Sequencing quality was assessed by FastQC and high-quality reads of libraries were trimmed using Trimmomatic (v0.39) prior to being mapped onto the mm10 mouse reference genome using STAR (v2.7.10b). Transcripts were quantified using StringTie (v1.3.6) and counts were imported to R using prep.de.py3 for normalization and differential expression analysis with DESeq2 (v1.42.0).

### ChIP-Seq analyses

Chromatin immunoprecipitation (ChIP) was performed as previously described in biological duplicates [23]. Briefly, control and KD MEF cell lines grown for RNA-seq analysis were stimulated with TNF-α at a concentration of 10 ng/mL for 30 min, washed with PBS once, crosslinked by incubating with disuccinimidyl glutarate (DSG) (ProteoChem) at a concentration of 2 mM in PBS for 30 min at room temperature with gentle rocking, and further crosslinked with 1% (vol/vol) formaldehyde in PBS for 10 min at room temperature. Crosslinking reactions were quenched by incubating with 125 mM final concentration of glycine at room temperature for 5 min. Cells were washed twice with ice-cold PBS and harvested by scraping. Pelleted cells were gently resuspended in 1 mL of PBS containing 0.5% NP-40 and 0.5 mM PMSF, and incubated in a rotary shaker for 10 min at 4°C. Nuclei were pelleted by centrifugation at 400 × *g* for 5 min at 4°C, washed once by resuspending in PBS containing 0.5% NP-40, then washed with PBS, followed by resuspension in 1 mL of RIPA buffer [25 mM Tris–HCl, pH 7.5, 150 mM NaCl, 0.1% SDS, 0.1% sodium deoxycholate, 1% NP-40, 1 mM ethylenediamine tetraacetic acid (EDTA), 1 mM DTT, and 0.5 mM PMSF]. Chromatin was sonicated using a Model 120 sonic dismembrator (Fisher) with 2 mm diameter tip with settings: 15 total cycles, 20-s pulse intervals with 40 s dwell time, and 80% amplitude to obtain fragments in general range of 200–1000 bp. Chromatin samples were centrifuged at 13 000 × *g* for 10 min at 4°C to remove debris and larger fragments. Supernatant containing fragmented chromatin of appropriate size range was diluted 1:1 with buffer containing 25 mM Tris–HCl, pH 7.5, 150 mM NaCl, 1 mM EDTA, 1 mM DTT, and 0.5 mM PMSF prior to immunoprecipitation steps. For immunoprecipitation, 30 μL of Protein G Magnetic beads (NEB) was mixed with 1 μL of anti-RelA antibody (Diagenode) in PBS containing 0.5% (m/v) bovine serum albumin (BSA). Beads were washed thrice with PBS before resuspending in binding buffer containing 25 mM Tris–HCl, pH 7.5, 150 mM NaCl, 0.05% SDS, 0.05% sodium deoxycholate, 0.5% NP-40, 1 mM EDTA, and 1 mM DTT. Sonicated chromatin was then added and samples were incubated overnight in a rotary shaker at 4°C. Beads were washed in following buffers, with each step 2 min long at room temperature in a rotary shaker unless otherwise specified: 3× with binding buffer, 3× with buffer containing 10 mM Tris–HCl, pH 7.5, 250 mM LiCl, 1% NP-40, 0.7% sodium deoxycholate, and 1 mM EDTA, 2× with buffer containing 10 mM Tris–HCl, pH 8.0, 1 mM EDTA, and 0.2% Tween 20, and 2× with buffer containing 10 mM Tris–HCl, pH 8.0, 0.1 mM EDTA, and 0.05% Tween 20. Beads were then resuspended in 25 μL of buffer containing 10 mM Tris–HCl, pH 8.0, and 0.05% Tween 20. Libraries were prepared on beads using NEBNext Ultra II DNA Library Prep kit (NEB) following manufacturer’s recommendations with 0.6 μM of KAPA Unique Dual-Indexed adapters (Roche). To each reaction of 46.5 μL, we added 16 μL of nuclease-free water, 4 μL of 10% SDS, 4.5 μL of 5 M NaCl, 3 μL of 0.5 M EDTA, 4 μL of 0.2 M EGTA, 1 μL of 20 mg/mL Proteinase K (NEB), and 1 μL of 10 mg/mL RNase A (Qiagen). The samples were incubated at 55°C for 1 h for Proteinase K and RNase A digestion, followed by incubation overnight at 65°C to reverse crosslinking. DNA was purified using 50 μL of Ampure XP beads (Beckman Coulter) and 20% PEG8000/2.5 M NaCl at a final concentration of 12% PEG and eluted in 13 μL of 10 mM Tris–HCl, pH 8.0, and 0.05% Tween 20. The eluted DNA was PCR amplified with KAPA HiFi 2× HotStart ReadyMix (Roche) and 10× Library Amplification Ready Mix primers (Roche) for 16 cycles. Amplified DNA libraries were then purified with Ampure XP beads at a 1:1 ratio (10% PEG final), analyzed by TapeStation (Agilent), and quantified by dsDNA Qubit (ThermoFisher). Pooled library DNAs of a size range of 200–500 bp were selected with Pippin HT (Sage Science) and 50 bp paired-end sequenced on the NovaSeq X Plus (Illumina).

ChIP-Seq analysis was performed as previously described [29]. Sequencing quality was first assessed by FastQC and libraries with high-quality reads were trimmed with Trimmomatic (v0.39). Trimmed FASTQ files were mapped to the mm10 mouse reference genome using Bowtie2 (v2.5.1). Peaks were then identified using HOMER v4.11.1 (findPeaks) with the parameters “-style factor -L 0 -C 0 -fdr 0.9 -minDist 200 -size 200 -o auto.” Raw tag counts were quantified with HOMER (annotatepeaks.pl) and tag counts were averaged between experimental replicates. Motif enrichment analysis was performed using HOMER (findMotifsGenome.pl) with the parameter “-size 200.” For gene ontology analysis, induced RelA peaks passing a threshold of log_2_ fold change (FC) > 1 and *P*-value < .05 were analyzed with the gseGO function from clusterProfiler (v4.8.3) with the org.Mm.eg.db (v3.17.0). Results were annotated along the ontology of biological processes with the following parameters: nperm = 10 000, pvalueCutoff = 0.05, and pAdjustMethod = “none.”

### Luciferase reporter assay

Complementary primers of specific promoters were mixed at a final concentration of 2 μM in buffer containing 10 mM Tris–HCl, pH 7.5, 50 mM NaCl, and 1 mM EDTA and annealed by placing them in beaker with 95°C water that was gradually cooled to room temperature. Annealed promoters were cloned within SalI and BamHI sites of the CMXTK-Luciferase vector (obtained from Dr D. Chakravarti, Northwestern University Feinberg School of Medicine).

HEK 293T were plated in 24-well plates 24 h prior to transfection so that they reached a confluence of ∼70%–80% at the time of transfection. Cells were transiently transfected using PEI with indicated reporters (e.g. in Fig. 2E) along with HA-RelA (1-551) or a control empty HA-vector, and Renilla luciferase expression vector under control of a CMV promoter. Cells were harvested 24 h post-transfection by scraping in 1× passive lysis buffer (Promega), and lysate was clarified by centrifugation at 13 000 × *g* for 10 min at 4°C. Luciferase activity was measured using the Dual-Luciferase Reporter Assay System (Promega). Readings were first normalized by comparing with activity of Renilla luciferase. The effect of RelA was then calculated by comparing samples transfected with HA-RelA versus empty HA-vector. For each case, three or more biological replicates were performed, and the results are represented as mean ± standard deviations (SD).

For luciferase reporter activity using HeLa cells (e.g. in Fig. 6B–E), the cells were also plated in 24-well plates 24 h prior to transfection so that they reached a confluence of ∼70%–80% at the time of transfection. Cells were transfected using PEI with indicated luciferase reporter construct, (or an E^MT^-P^MT^ construct—activity from which was later used for normalization), along with either FLAG-tagged cofactor or an empty FLAG vector (as a control) for 48 h, followed by treatment with 10 ng/mL TNF-α for 6 h prior to harvest by scraping in 1× passive lysis buffer (Promega). Cell lysate was clarified by centrifugation at 13 000 × *g* for 10 min at 4°C. Luciferase readings were first normalized by lysate protein concentration measured by Bradford reagent. Fold-changes in activity from the luciferase reporter (compared to activity from E^MT^-P^MT^) in the presence or absence of cofactor were then analyzed. Data represented are mean ± SD of three or more biological replicates.

### Electrophoretic mobility shift assay

Oligonucleotides were ^32^P-end labeled with [γ-^32^P]ATP (PerkinElmer) using T4-polynucleotide kinase (New England Biolabs). Labeled DNA was purified of free [γ-^32^P]ATP using a Microspin G-25 column (Cytiva) and annealed with complementary DNA as described in luciferase reporter assay. These radiolabeled DNAs were incubated with different amounts of recombinant RelA:RelA or p50:RelA at room temperature for 15 min in binding buffer containing 10 mM Tris–HCl, pH 7.5, 50 mM NaCl, 10% glycerol, 1% NP-40, 1 mM EDTA, and 0.1 mg/mL sonicated salmon sperm DNA (Fisher). Recombinant proteins of different concentrations were prepared using dilution buffer containing 20 mM Tris–HCl, pH 7.5, 50 mM NaCl, 10% glycerol, 1 mM DTT, and 0.2 mg/mL BSA for the reaction mixtures. Complexes were separated by electrophoresis at 200 V for 1 h at room temperature in a pre-run 5% non-denaturing polyacrylamide gel in 25 mM Tris, 190 mM glycine, and 1 mM EDTA buffer. Gels were dried, exposed on a phosphor screen overnight, and shifted bands were scanned by Typhoon FLA 9000 imager (Cytiva) for analysis.

For EMSA with nuclear RelA, MEF cells were harvested by scraping in PBS and lysed in cytoplasmic lysis buffer consisting of PBS with 0.5% NP-40 and 0.5 mM PMSF. Nuclei were pelleted by centrifugation at 300 × *g*, washed twice with PBS, and resuspended in extraction buffer containing 25 mM Tris–HCl, pH 7.5, 420 mM NaCl, 10% glycerol, 0.2 mM EDTA, 1 mM DTT, and 0.5 mM PMSF to obtain nuclear extract. Seven micrograms of nuclear extract (quantified by Bradford assay) was mixed with radiolabeled DNA (final concentration ∼2 nM) in a binding reaction of 10 μl consisting of 25 mM Tris–HCl, pH 7.5, 150 mM NaCl, 10% glycerol, 1 mM EDTA, and 0.1 mg/mL sonicated salmon sperm DNA. Complexes were analyzed by non-denaturing gel electrophoresis as outlined earlier.

### Real-Time qPCR of RNA samples

Total RNA of cultured cells was isolated using TRIzol reagent (Invitrogen) followed by isopropanol precipitation, and RNA concentration was determined in ND-1000 spectrophotometer (Nanodrop). Complementary DNA (cDNA) was synthesized with 1 μg of total RNA using Maxima H Minus Master Mix (Thermo Fisher) in a reaction volume of 5 μL. Synthesized cDNA was then diluted four-fold with nuclease-free water and 1 μL sample was used as a template for real-time qPCR using Luna Master Mix (NEB) on a Bio-Rad CFX Connect Real-Time PCR detection system. The list of primers used for reverse transcription quantitative polymerase chain reaction (RT-qPCR) is listed in Supplementary Table S1. Expression of housekeeping gene *Gapdh* was used as the control for normalization. Normalized expression was calculated using the ΔCt method by subtracting the mean cycle threshold (Ct) of the target gene from the average Ct of *Gapdh*. Normalized expression was calculated as 2^-ΔCt^ from three independent experiments and is represented as mean ± SD.

### DNA pulldown and mass spectrometry analyses

For pulldown of proteins with streptavidin-DNA, 5′ biotinylated oligonucleotides (IDT) were annealed with corresponding nonbiotinylated reverse complement oligonucleotides at a final concentration of 45 μM. For each pulldown reaction, 40 μL of 45 μM annealed DNA was mixed with 100 μL of Neutravidin agarose resin (Thermo Fisher) in a total volume of 400 μL in a final buffer containing 10 mM Tris–HCl, pH 7.5, 50 mM NaCl, and 0.5 mM EDTA. All centrifugation steps were performed at 3000 × *g* for 1 min at 4°C. Biotinylated DNAs were immobilized onto beads by gentle mixing in a rotary shaker for 1 h at room temperature. Beads were washed extensively with the pulldown buffer containing 25 mM Tris–HCl, pH 7.5, 150 mM NaCl, 5% glycerol, 0.1% NP-40, and 1 mM DTT, and finally resuspended in a 50 μL pulldown buffer. Separately, the nuclear extract of TNF-α (20 ng/mL for 30 min)-treated MEF cells was prepared as previously outlined. Approximately 250 μg of nuclear extract was diluted 2.8-fold with the pulldown buffer without NaCl but supplemented with 0.5 mM PMSF to reduce the final NaCl concentration from 420 to 150 mM. The diluted nuclear extract was first precleared by gently mixing 50 μL of the biotinylated double mutant DNA (2DM) immobilized to streptavidin beads at 4°C for 2 h. The mutant beads were then pelleted and discarded, and the precleared lysate was then mixed with the corresponding experimental beads gently in a rotary shaker overnight at 4°C. These beads were then washed 4 times with a 1 mL volume of pulldown buffer at 4°C. Biological duplicates of each unique pulldown experiment were performed.

Microcapillary LC/MS/MS was performed on the bead eluant for identification of peptides at the Taplin Biological Mass Spectrometry Facility at the Harvard Medical School. Beads were first washed with 50 mM ammonium bicarbonate. Then it was incubated with 100 μL of 200 ng/μL modified sequencing grade trypsin (Promega) at 37°C overnight. Beads were discarded by centrifugation and the supernatant was dried in a speed-vac for 1 h. These dried samples were then resuspended in 50 μL of HPLC solvent A (2.5% acetonitrile, 0.1% formic acid), desalted by STAGE tip, and loaded onto a nanoscale reverse-phase HPLC capillary column of 2.6 μm C18 spherical silica beads (100 μm inner diameter × 30 cm length) pre-equilibrated with solvent A. Peptides were eluted with an increasing acetonitrile gradient using solvent B (97.5% acetonitrile, 0.1% formic acid), and the eluted peptides were electrospray ionized prior to entering a Velos Orbitrap Elite ion-trap mass spectrometer (Thermo Fisher). Peptides were detected, isolated, and fragmented to produce a tandem mass spectrum of specific fragment ions for each peptide.

Fragmentation pattern of peptides was analyzed with the program Sequest (Thermo Fisher) against the UniProt database (https://www.uniprot.org/taxonomy/10090) in order to determine the peptide sequences and protein identity. The query databases included reversed sequence entries, and the data were filtered using a peptide false discovery rate (FDR) of 1%. Total peptide counts were used to compare samples, and peptides with a total count fewer than five were excluded. Peptide counts of these selected proteins were first normalized for each individual pulldown experiment by dividing by the sum total of peptides. These normalized counts were used to calculate the *Z*-score value for each protein. The *Z*-score values were then used to filter noise and select proteins that are enriched in pulldown with biotin-Cxcl2 DNA or biotin-Ccl2 DNA compared to the biotin-control DNA (average *Z*-score of Cxcl2 and Ccl2 pulldowns >0.5 compared to control DM pulldown). The gene set enrichment analysis was performed using the gene ontology of biological process database with the gseGO function from clusterProfiler (v4.10.0) with the org.Mm.eg.db (v3.18.0) with the following parameters: nPerm = 10 000, pvalueCutoff = 0.05, minGSSize = 3, maxGSSize = 300, and pAdjustMethod = “none.” The chord plot was generated using the GOChord function from GOplot (v1.0.2).

### Biolayer interferometry assay and analysis

The assay was performed in Octet K2 system (ForteBio) and protocol was adapted based on Li *et al.* [13]. Briefly, biotinylated oligonucleotides were mixed with complementary oligonucleotides at a ratio of 1:1.2 in buffer containing 10 mM Tris–HCl, pH 7.5, 50 mM NaCl, and 1 mM EDTA and annealed by placing samples in beaker with 95°C water that was gradually cooled to room temperature, and then placed in ice. Hydrated Octet Streptavidin (SA) biosensors (Sartorius) were loaded with these DNA duplexes by placing them in 200 nM of DNA for 10 s in BLI buffer consisting of 25 mM Tris–HCl, pH 7.5, 150 mM NaCl, 0.02% Tween 20, 0.1 mg/mL sonicated salmon sperm DNA, and 1 mM DTT. A reference for background subtraction is always generated using a sensor that was not loaded with DNA. For all assays, a baseline was first obtained by incubating sensors in BLI buffer for 60 s. An association phase of 120 s was observed by dipping the sensor-biotinylated DNA probe in 12.5, 25, 50, 100, or 200 nM of recombinant His-RelA (1-551), followed by a dissociation phase of 180 s by placing the sensor back in BLI buffer without protein. For regeneration of DNA-bound sensor (i.e. to remove the bound protein), it was placed in BLI buffer containing 1 M NaCl for 5 s, followed by 3× washes of 5 s in BLI buffer. Data were processed using Sigmaplot 15 to observe the binding kinetics and obtain affinity values.

### Sanger sequencing

CRISPR-modified MEF cell lines were harvested by scraping, and genomic DNA was collected using phenol:chloroform:isoamyl alcohol (25:24:1; Thermo Fisher) and ethanol precipitation. The DNA pellet was resuspended in TE buffer and used as a template for PCR reactions using Taq polymerase (NEB) with primers targeting the Cxcl2 promoter (Supplementary Table S1). The completed PCR reaction was separated by agarose gel electrophoresis and visualized with ethidium bromide. The band corresponding to the expected 200 bp Cxcl2 promoter amplicon was excised with a sterile razor and purified using the Monarch DNA Gel Extraction kit (NEB) following manufacturer’s specifications. Approximately 10 ng of purified PCR product was then subject to Sanger sequencing (Genewiz) in both directions using either the forward or reverse primer.

### Immunoprecipitation assay

For FLAG pulldown assays, HEK293T cells were plated in six-well culture dishes (GenClone) at 80% confluence and transfected using PEI with HA-tagged RelA and either empty vector or FLAG-tagged Nfatc1, Nfat5, Tead3, Cux1, or Smad4 overexpression constructs for 24 h. Cells were then stimulated with mouse TNF-α (BioBharati) for 30 min, washed once with ice-cold PBS, and lysed by direct application of lysis buffer containing 25 mM Tris–HCl, pH 7.5, 150 mM NaCl, 5% glycerol, 1% NP-40, 1 mM DTT, and 0.25 mM PMSF to the well. Lysate was clarified by centrifugation at 13 000 × *g* for 15 min at 4°C. Anti-FLAG M2 agarose beads (Sigma) were incubated with the lysate for 2 h at 4°C in a gentle rotary shaker. Beads were washed extensively with lysis buffer, and 4× SDS gel loading dye was added to a final concentration of 1× prior to heating at 95°C in a heat block for 5 min. Samples were separated on a 10% sodium dodecyl sulfate–polyacrylamide gel electrophoresis (SDS–PAGE) gel at 200 V for 45 min and transferred to nitrocellulose membrane for western blot analysis.

## Results

### Clusters of κB sites of a wide range in affinity are present in promoters of NF-κB target genes and these collectively dictate recruitment of RelA to DNA

**Figure 1.**
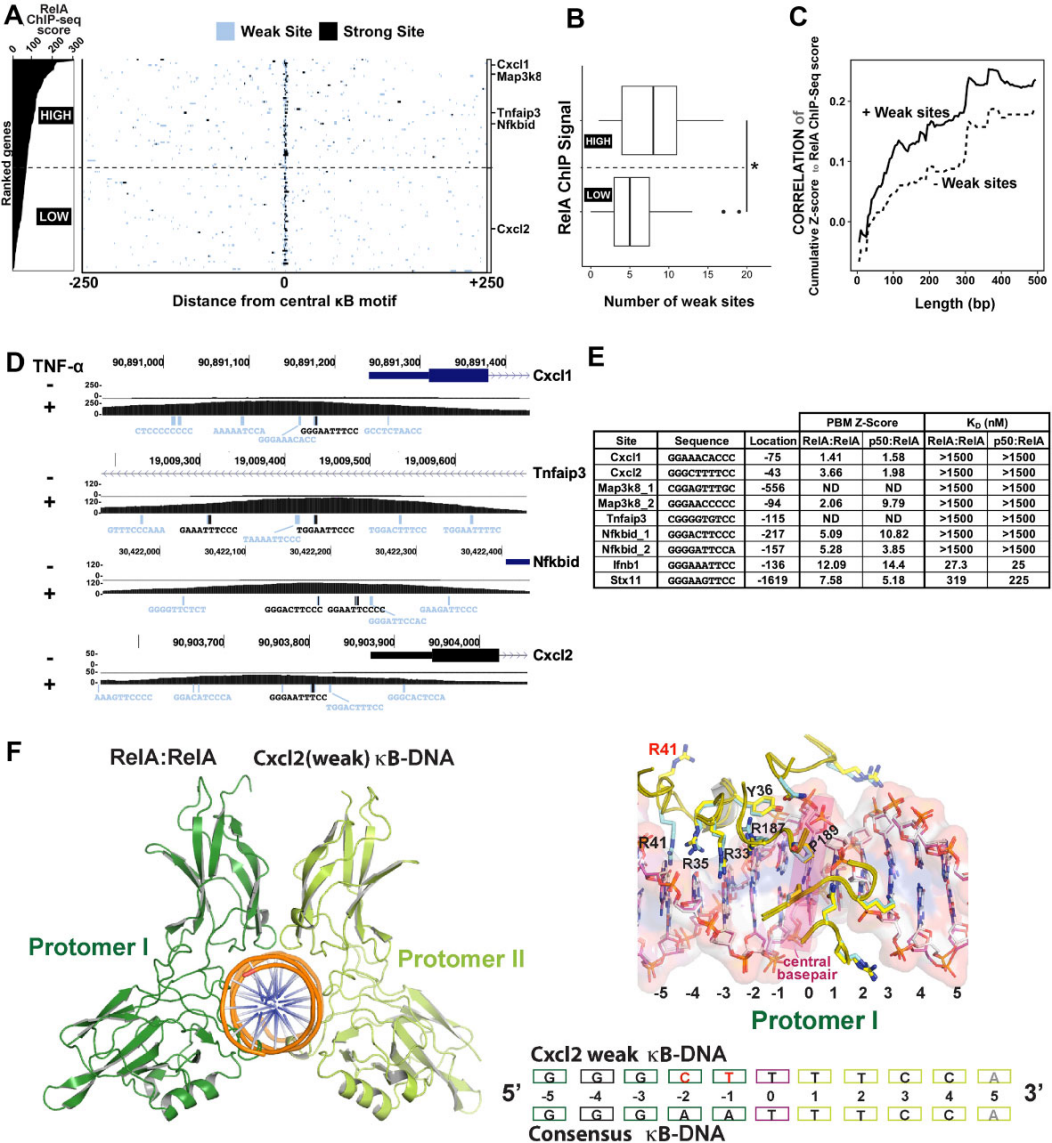
Identification and analysis of weak κB motifs in RelA-binding promoter sites of TNF-α-responsive genes. (**A**) Left panel, genes ranked based on RelA ChIP-seq scores in the analyzed promoter regions (separated by a dotted horizontal line in two halves for high and low). Right panel, 500-bp regions centered around the strongest RelA motif within RelA ChIP-seq peaks (Ngo *et al.* 2020) were analyzed for the presence of strong (black) and weak (blue) κB motifs, as defined by a cut-off *Z*-score of 6 in PBM analysis of p50:RelA heterodimer (Siggers *et al.* 2009). *n* = 116 RelA peaks analyzed. (**B**) Boxplot of number of weak κB sites in the top half versus bottom half of genes ranked by ChIP-Seq score. *P < .05. *P*-value was calculated by two-tailed Student’s *t*-test. (**C**) Dependence of the “Pearson’s correlation between RelA ChIP-seq score and cumulative promoter *Z*-score surrounding the central κB site” on “analysis window length” of 10–500 bp. Analysis either included (solid) or excluded (dashed) contribution of weak κB motifs. (**D**) Genome browser tracks showing κB motifs and their sequences within a 500 bp window of Cxcl1, Cxcl2, Tnfaip3, and Nfkbid promoters, along with TNF-α induced RelA ChIP-seq signal. Strong and weak κB site sequences in black and blue, respectively. RelA ChIP-seq signal intensity is indicated on the left. (**E**) BioLayer interferometry assay derived equilibrium binding affinity (primarily from this study) with *Z*-scores (Siggers *et al.* 2009) of κB sites for RelA homodimer and p50:RelA heterodimer. (**F**) A cartoon representation of the RelA homodimer (left) bound to a weak κB site derived from Cxcl2 gene (bottom). Structural similarity/dissimilarities in interaction of the RelA protomers with weak versus strong κB motifs (right).

We examined the relationship between ChIP-seq scores of RelA, reflecting occupancy of RelA on DNA in the cell, and sequence of κB sites within the promoters of over 100 NF-κB-regulated murine genes that are stimulated by TNF-α within a 30-min stimulation period, as reported by Ngo *et al.* [30]. The sites are designated as κB sites whether they matched any of the 1800 sites with a measurable *Z*-score derived from an earlier protein microarray-based assay (PBM) analysis [14]. We characterized the strong and weak κB sites of the promoter and enhancer regions to assess how a set of these κB sites could facilitate transcription through recruitment of RelA dimer in association with accessory cofactors. The κB sites were designated as strong or weak considering both their physical and functional characteristics. Binding characteristics of A-centric (IFNβ-κB) and G-centric (STX11-κB) κB sites toward p50:RelA heterodimer or RelA:RelA homodimer were used as benchmarks [13]. In general, κB-sites with affinity (K_D_) to NF-κB RelA dimers above and below 100 nM—assessed *in vitro* using a solution-based binding assay at near-physiological ionic strength and pH—were defined as weak and strong, respectively. The Z score of binding of 1800 κB sites to NF-κB RelA dimer estimated in the PBM assay [14] was tallied to it, and an arbitrary Z score threshold of ∼6.0 separated weak and strong κB sites. Additionally, a transcriptional activation potential of a single κB site in luciferase reporter-based assay of less or more than two-fold was considered functionally weak and strong, respectively. The spectrum of considered κB sites deviated from the 10-bp consensus at most at three positions. Strikingly, we observed that not all strong κB sites are aligned with RelA ChIP peaks. Rather, we observed that the 500-bp window centered around the ChIP-seq peak for most TNF-α-responsive genes contained a strong κB site surrounded by multiple weak κB sites (Fig. 1A and Supplementary Table S2). Analysis of the weak κB site abundance around RelA ChIP-seq peaks sorted by intensity (ranked as top and bottom) revealed a significantly higher number of weak κB sites around top-ranked RelA peaks (Fig. 1B). However, we observed only a weak correlation between RelA ChIP-seq score and the *Z*-score of the strong κB site proximal to the central peak motif (Supplementary Fig. h S1A). This suggested the possibility that individual affinity of the central strong κB site is insufficient in predicting occupancy of RelA dimers to promoter regions in cells. Intriguingly, we observed a stronger correlation of ChIP-seq strength with calculated combined *Z*-score of sites in an extended window of up to 500 bp around the central strong κB motif (Supplementary Fig. S1A). The correlation reduced with exclusion of the contribution from weak κB motifs, suggesting a significant contribution of the colocalized weak κB motifs in recruitment of RelA (Fig. 1C). The clusters of κB sites primarily spanned ∼150 to 200 bp within the analyzed window of 500 bp, thus mimicking the span of an open chromatin observed within promoters and enhancers during transcription [31].

We further analyzed the arrangement of κB sites in promoters of prominent NF-κB-responsive murine genes Cxcl1, Cxcl2, Nfkbid, and Tnfaip3 (Fig. 1D). Both Cxcl1 and Cxcl2 contain an identical strong site (GGGAAATTCC) but the overall ChIP score at the Cxcl2 promoter is noticeably lower than that at Cxcl1 (250 versus 50). This may be reflective of a higher number of weak κB sites in the Cxcl1 promoter compared to Cxcl2 promoter (13 versus 8). The composite cumulative *Z*-score of Cxcl1 weak sites for RelA dimers is also higher than that of Cxcl2 (70.6 versus 58.5). Some of these weak sites, such as GGATTTTTT and GGGAATTTTA (deviations from consensus are underlined), found near the strong site in Cxcl1 and Tnfaip3 promoters, displayed minimal resemblance to the κB consensus. To assess whether these putative weak sites engage RelA dimers, we measured affinities of selected κB sites for both NF-κB RelA homodimer and p50:RelA heterodimer (of WT full-length proteins) at a near-physiological salt concentration by biolayer interferometry (BLI) assays. Both NF-κB dimers bound these putative sites with a much-reduced affinity when compared with binding to IFN-β1: GGGAAATTCC (K_D_ ∼27 and *Z*-score ∼14) and STX11: GGGAAGTTCC (K_D_ ∼250 and *Z*-score 5.8) sites as references of strong and weak κB sites. Although minor binding was detected for most weak sites at higher protein concentrations, precise K_D_ values could not be determined. Two of the sites tested did not display any binding even at the highest protein concentration (Fig. 1E and Supplementary Fig. S1B) and these are estimated to have K_D_> 1.5 μM. Since the nuclear concentration of RelA upon TNF-α stimulation is observed to be around 200–250 nM (Supplementary Fig. S1C), only about 6% of these dimers are projected to bind if K_D_ is around 3.0 μM. These findings raised queries on the authenticity and functional relevance of putative weak sites *in vivo*. It should be noted that there is a discrepancy between binding constants measured with native proteins at near physiological ionic strengths using solution-based methods versus truncated proteins under non-physiological ionic strengths using microarray-based methods. We also qualitatively examined the binding strengths of weak and strong sites within the Cxcl2 promoter using EMSA with recombinant p50:RelA heterodimer and RelA:RelA homodimer (Supplementary Fig. S1D). The results indicated that mutation of both κB sites in Cxcl2 promoter, referred to here as Cxcl2 double mutant (2DM), abrogated binding of NF-κB completely, while mutation of only the weak site (2ΔW) did not cause any discernible effect. Sequence-specific binding at the weak κB site (2ΔS) was retained upon mutation of strong site for both dimers, demonstrating that NF-κB has the capacity to bind this weak motif. Collectively, these findings led us to conclude that the putative weak sites with detectable binding *in vitro* may have a functional role.

To further evaluate whether the binding by NF-κB dimers to weak κB sites is specific and authentic, we determined the crystal structure of RelA homodimer in complex with a weak κB site (^−5^G^−4^G^−3^G^−2^ C^−1^ T^0^T^+1^T^+2^T^+3^C^+4^C) present in the promoter of the Cxcl2 gene (the non-cognate base pairs are underlined and the pseudo-dyad axis separating the two halves of the κB site is marked ^0^T) (Supplementary Table S3). The structure revealed that the binding interaction of RelA dimer to this weak site is remarkably similar to that observed with consensus κB sites (Fig. 1F; PDB code 9E6W). The Arg33 and Arg35 engaged to the ^−4^G^−3^G of one half-site, as predicted. However, the presence of non-cognate bases (^−2^C^−1^T compared to ^−2^A^−1^A, the cognate bases for the RelA homodimer) influenced the global DNA architecture such that Arg187 is positioned slightly differently, but more importantly, R41 could not engage with ^−5^G of this weak site. This altered protein–DNA contact likely entails the weaker interaction, which nonetheless is specific. Taken together, these results reflect an enrichment of weak κB motifs in the regulatory regions of NF-κB target genes, which can indeed be recognized specifically by RelA; however, a more elaborate mechanism must underlie an efficient occupancy of these weaker sites.

### Weak sites within κB clusters drive NF-κB-induced transcription

**Figure 2.**
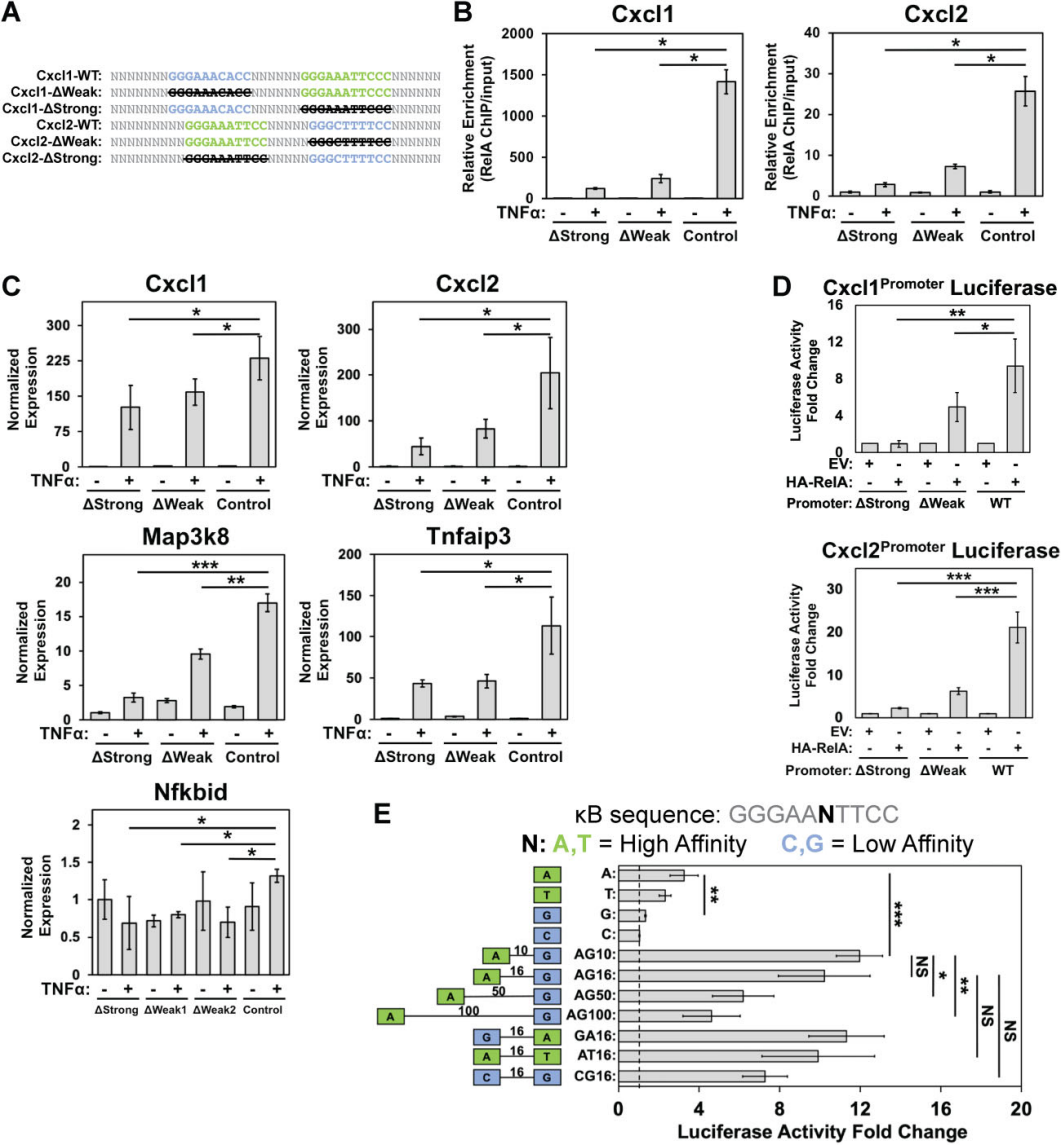
Multiplicity of κB sites (strong and weak) influences recruitment of RelA and transcription. (**A**) A schematic of the arrangements of strong and weak κB sites in Cxcl1 and Cxcl2 promoter regions targeted individually by CRISPR-mediated deletion. Separately, a nearby off-target site of the promoter was also mutated using CRISPR to be used as a gene-specific control. (**B**) Effect of deletion of strong, weak, or non-κB site (control) of Cxcl1 and Cxcl2 promoters on RelA ChIP-qPCR upon 30-min TNF-α stimulation. Data are normalized by amount of input DNA, and changes represented are relative to promoter with deletion of control non-κB site. *P < .001. *P*-value was calculated by two-tailed Student’s *t*-test. *n* = 3 independent experimental replicates. Error bars represent SD. (**C**) Normalized transcript levels of Cxcl1, Cxcl2, Tnfaip3, Map3k8, and Nfkbid in MEF cells upon 1 h TNF-α stimulation measured by RT-qPCR following deletion of a strong, weak, or control site. Data represented are normalized by Gapdh expression. *P < .05, ** *P* < .001, *** *P* < .0001. *P*-value was calculated by one-tailed Student’s *t*-test. For all data points, *n* = 3 experimental replicates. Error bars represent SD. (**D**) Expression of luciferase reporter plasmids driven by Cxcl1 or Cxcl2 promoter (WT or mutant) regions in HEK 293T cells co-transfected with HA-tagged RelA or control HA-tagged EV. Luciferase readings are normalized with control Renilla luciferase activity, and fold-change is relative to EV for a given luciferase construct. *P < .05, **P < .01, *** *P* < .0001. *P*-value was calculated by one-tailed Student’s *t*-test. For all data points, *n* = 3 experimental replicates. Error bars represent SD. (**E**) Expression of luciferase reporter plasmids under various synthetic promoters (containing single or combinations of differentially placed strong and weak κB sites) in HEK293T cells co-transfected with HA-tagged RelA expression vector. Numbers within schematic on left represent number of bp separating κB sites. Luciferase readings are normalized to control Renilla luciferase activity, and fold change is relative to control EV for the given luciferase construct. *P < .05, **P < .01, *** *P* < .005. *P*-value was calculated by one-tailed Student’s *t*-test. For all data points, *n* = 3 experimental replicates. Error bars represent SD.

We followed through next by exploring the potential of above-mentioned weak κB sites in activating transcription of NF-κB target genes in cellular milieu. We selectively deleted strong and its neighboring weak κB sites, independently, in the promoters of NF-κB target genes Cxcl1, Cxcl2, Nfkbid, Map3k8, and Tnfaip3 of MEF cells using CRISPR–Cas9 genome editing technology (Fig. 2A and B). The placement of weak sites in different genes was different in sequence, orientation, and relative distance from the strong site. Control cells were established by deletion of a random segment away from any κB site, and sequencing of bulk populations indicated the deletions to be averaging around 4–10 bp (Supplementary Fig. S2). These pooled edited cell lines were grown, and the transcript levels of target genes were measured by qPCR upon treatment with TNFα (10 ng/ml) for 1 h. We observed a significantly reduced level of transcription in all five target genes upon deletion of either the strong or weak sites (Fig. 2B). To assess whether deletion of weak or strong sites in the promoter of one gene could affect the expression of other genes, we analyzed the expression levels of Cxcl2, Map3k8, and Tnfip3 following deletion of κB sites in the Cxcl1 promoter, and analyzed expression of Cxcl1, Cxcl2, and Map3k8 upon deletion of κB sites in the Tnfaip3 promoter. For the most part, deletion of κB sites in genes within one chromosome did not alter the expression of genes located on others. However, we observed that deletion of either a strong or weak κB site in the promoter of Cxcl1 altered the expression of Cxcl2 (Supplementary Fig. S2B). These two genes are present in tandem on the same chromosome (chr 4 in humans and chr 5 in mice). Thus, these genes appear to be regulated by the overlapping enhancers, suggesting a potential for local regulatory interactions. ChIP qPCR assays using a specific antibody against RelA with the WT and mutated Cxcl1 and Cxcl2 promoters indicated a significant reduction of RelA ChIP signal upon deletion of κB sites, weak or strong, compared to a control site deletion (Fig. 2C). We are unclear as to why the mutant weak site had an equally strong effect as the mutant strong site in case of Tnfaip3. This further indicates that weak sites are not always redundant, and one possibility is that this weak site strongly engages another factor critical to RelA recruitment. These results support a critical function of weak sites in recruitment of NF-κB RelA to promoter and in transcription.

To further support the authenticity and functionality of these weak sites in transcription, we assessed expression potential of promoter segments from Cxcl1 and Cxcl2 genes (as used in EMSA in Supplementary Fig. S1D) using luciferase reporter constructs (Supplementary Table S1). Mutation of either the strong or the weak site led to a drastic reduction in reporter expression, similar to that observed in the cellular context. This provides further support to both strong and weak κB sites of the Cxcl1 or Cxcl2 promoters being critical in optimal expression and alludes to a functional synergy between the multiple sites during transcriptional activation (Fig. 2D and Supplementary Table S4). To analyze this synergy further, we assessed expression of reporters with synthetic promoters containing various combinations of strong and weak κB sites with different spacing and orientations. We not only observed that two sites act synergistically but also observed a strikingly robust reporter activity from two weak sites similar to that observed with two strong sites (Fig. 2E). Reporter activity decreased progressively as the spacing between two sites was increased to 100 bp. Overall, these observations reveal a strong activating influence of clusters of κB sites, strong or weak, in enhancing gene expression—outperforming the expression potential of a single strong site.

### A multitude of nuclear proteins associate with κB DNA-bound NF-κB upon inducing signal

**Figure 3.**
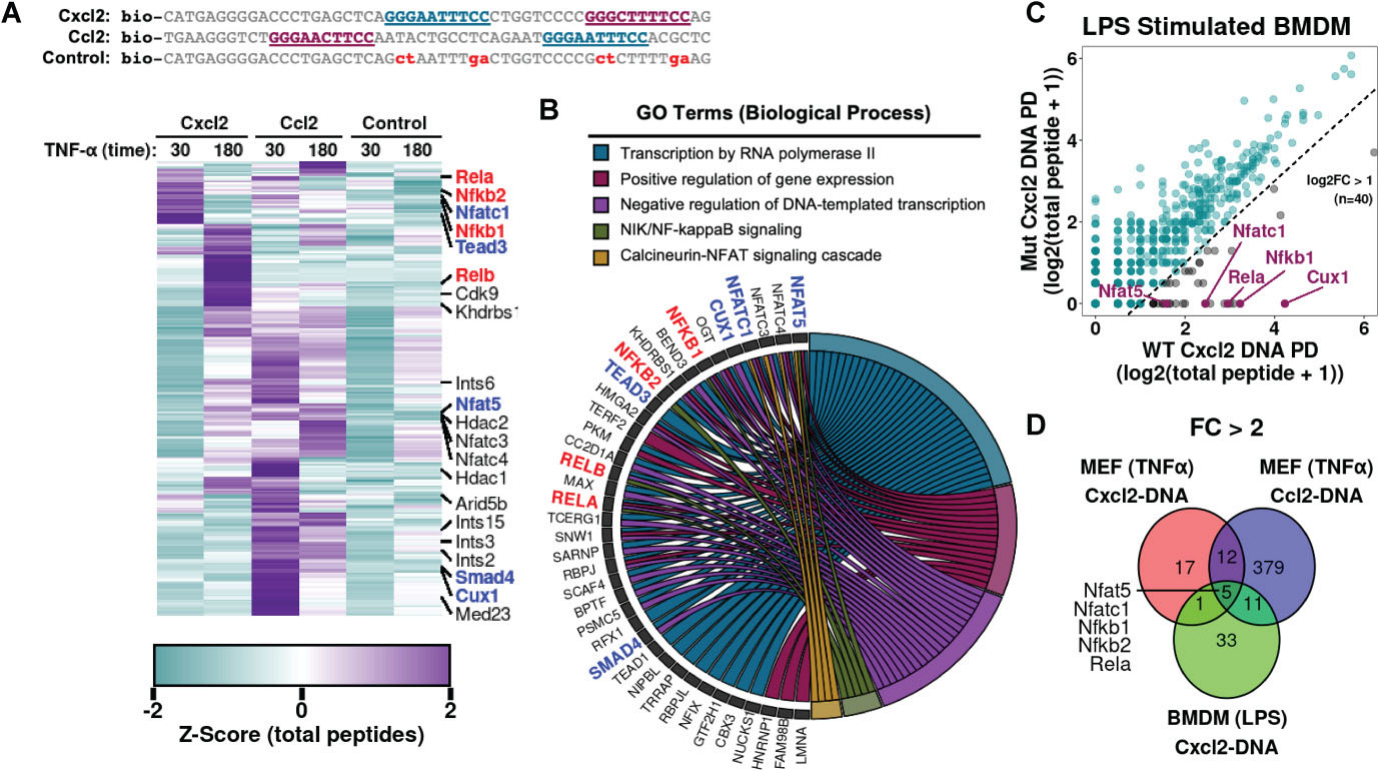
Association of a multitude of proteins to DNA adapted from tandem κB sites observed in RelA-responsive promoters of Cxcl2 and Ccl2 genes. (**A**) (Top) Sequence of biotinylated DNA fragments used in pulldown experiments—containing κB sites from promoter region of Cxcl2 or Ccl2 genes, or a control DNA with no κB sites. (Bottom) Heatmap representation of total peptide (k-means clustered) *Z*-scores with mass-spec of DNA-mediated pulled-down from nuclear extracts of TNF-α-treated MEF cells. Cells were stimulated with TNF-α for either 30 or 180 mins. Total peptides for each protein were normalized by total number of peptides identified in corresponding pulldown, and proteins presented represent average *Z*-score of Cxcl2 and Ccl2 >0.5 relative to the control pulldown. NF-κB members are annotated in red and the putative cofactors in blue. Data represent an average of *n* = 2 experimental replicates. (**B**) Chord diagram of biological pathway-specific gene ontology classification of enriched proteins identified in panel (A). (**C**) Correlation plot of total peptide counts from proteins identified by MS in nuclear extracts of LPS-treated BMDM enriched by binding to Cxcl2 DNA fragment compared to mutant DNA. Dotted line represents a log_2_FC of 1 for Cxcl2 DNA/mutant DNA. Data represent an average from *n* = 2 experimental replicates. (**D**) A Venn diagram displaying commonality and specificity of cofactors from different cell types, DNA, and stimuli.

The weak affinity of most κB sites found in promoter regions of RelA-responsive genes raised queries both about their functional authenticity and the possibility of disguised processes that could provide multisite cooperativity, rendering effective recruitment of RelA dimers to promoter elements *in vivo*. Since nucleosomes are known to gather a large array of nuclear factors, it is not far-fetched that additional nuclear factors could influence recruitment of RelA or other TFs in cells. To explore this, we investigated factors in nuclear extracts of MEF cells post 0.5- and 3-h TNF-α stimulation that associate with Cxcl2 promoter DNA fragment (sequence mimicking that of EMSA and reporter expression assays) biotinylated for pulldown assays. The pulled-down proteins were identified by mass spectrometry, and the specificity of factors was established by comparison of results with WT and a mutant (dκB; does not bind RelA since both κB sites are deleted) promoter fragments (Supplementary Fig. S3A). Several nuclear proteins (Fig. 3A and Supplementary Table S5), including other TFs, DNA-repair proteins, RNA-binding proteins, and metabolic enzymes, were observed. Various promiscuous/non-sequence-specific DNA-binding proteins were also observed. Among the TFs, members of the NFAT family were particularly notable, and those of TEAD, SMAD, and CUX families were well-represented. Among the RNA-binding proteins, the SR family splicing factors and hnRNPs were abundant. Significant difference of associated factors was observed in pull-downs with nuclear extracts of MEF cells isolated at 0.5 h versus 3 h post-TNFα stimulation. We surmise that this post-induction temporal dependence is likely due to significantly altered characteristics of proteome. The report of a large number of RelA-interacting proteins that includes several TFs in the whole cell extract has recently been reported [32]. To investigate whether association of these nuclear factors displays a dependence on promoter characteristics, we performed similar experiments with the Ccl2 promoter DNA fragment (Fig. 3A). The association of proteins to Cxcl2 and Ccl2 DNA fragments was largely similar, with a few exceptions. Pathway analysis revealed significant enrichment of proteins in transcriptional regulation, NF-κB signaling, and NFAT signaling pathways (Fig. 3B). Further investigation with extracts from LPS-treated bone marrow-derived macrophage (BMDM) cells corroborated pull-down of DNA-binding TFs, RNA-binding proteins, and various enzymes (Fig. 3C and Supplementary Table S5). Notably, NFAT factors were pulled down from extract of LPS-treated BMDM cells, as observed with extract of TNF-α-treated MEF cells.

We explored possible roles of other TFs by focusing on Nfatc1, Nfat5, Tead3, Cux1, and Smad4, all of which can regulate transcriptional programs through their own cognate sequences. These factors were observed in pulldown with both Cxcl2 and Ccl2 promoter elements with cellular extracts harvested from at least one of two post-induction time points. We tested direct interaction of factors (FLAG-fused) with RelA (HA-tagged) by pull-down to ascertain whether direct interaction of factors with RelA could regulate recruitment of RelA to specific promoters (Supplementary Fig. S3B). We observed association of RelA with all five factors, albeit of different strengths. Nfatc1 interacted with RelA the strongest and it could also pull down endogenous RelA efficiently (Supplementary Fig. S3C). We also observed that Nfatc1 localizes to the nucleus along with RelA upon TNF-α stimulation (Supplementary Fig. S3D). Using EMSA, we observed that affinity-purified Nfatc1 and Tead3 from HEK293 cell extract can bind to the WT Cxcl2 DNA probe (2WT), which contains both strong and weak κB binding sites. Binding was abolished when both sites were mutated (2DM)—similarly to that observed with NF-κB p50:RelA heterodimer (Supplementary Fig. S3E). We further performed EMSA to assess whether p50:RelA heterodimer can form a ternary complex with Nfatc1 or Tead3 on DNA. Observation of smeary, shifted bands together with a reduction of individual Nfatc1 and p50:RelA single complexes (Supplementary Fig. S3F, left) suggests a possible formation of new complexes. Ternary complexes were not observed with fragments containing only one κB site, either strong or weak, although Nfatc1 could still bind to both of these mutant probes singly. In contrast, no new bands were detected when Tead3 was included with p50:RelA on any of the three DNA probes. Surprisingly, Tead3 bound to the WT and mutant probe containing only the strong site, but not to the probe containing only the weak site—suggesting an overlap of Tead3 consensus site with only the strong κB site (Supplementary Fig. S3F, right). These findings suggest the possibility of Nfatc1 assembling on the Cxcl2 promoter on its own or simultaneously with NF-κB, in particular when multiple κB sites are present. In contrast, Tead3 appears to engage with the strong κB site, and the access appears to be competed out by NF-κB. Whether observed simultaneous interactions are cooperative or competitive or both and whether these are a general feature across other NF-κB target promoters remain to be probed. We speculate that a combined effect of cooperative and/or competitive interactions among RelA and RelA-promoter-associated TFs (termed TF-CoFs) facilitates recruitment of RelA to promoters.

### TF-cofactors support recruitment of NF-κB RelA to promoter

To test whether binding of RelA to the κB sites is influenced by Nfatc1, Nfat5, Tead3, Cux1, and Smad4, we created shRNA-mediated MEF KD cell lines. A general reduction of ∼60%–80% in protein levels was observed in KD cells when compared with a control MEF cell (scramble-KD) that underwent a similar KD procedure with a scrambled sequence (Supplementary Fig. S4A). The amounts of total RelA in these KD cells were similar to that observed in scramble-KD MEF (Supplementary Fig. S4B).

**Figure 4.**
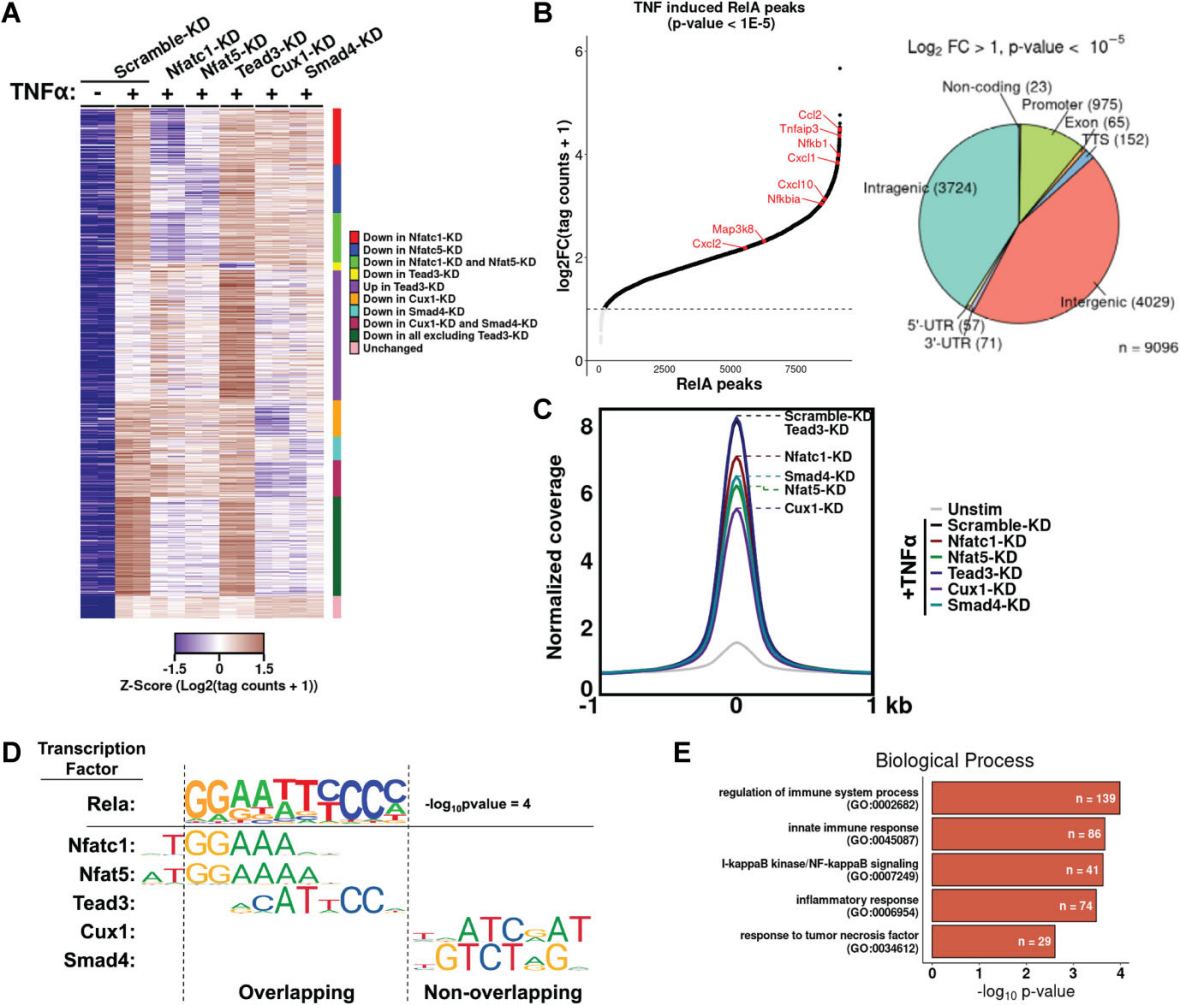
ChIP-seq indicating involvement of TF-cofactors in NF-κB RelA recruitment. (**A**) Heatmap representation of genome-wide RelA binding *Z*-score from ChIP-Seq in TNF-α stimulated (30-min) KD cell lines. Peaks were filtered by TNF-α-induced change in signals (log_2_FC > 1, *P*-value < .05, *n* = 2078 peaks in Scramble-KD cells). Colors represent *Z*-score for each peak and categories of clustering are listed on the right. *n* = 2 biological replicates for all samples. (**B**) (Left) Plot of ranked RelA-induced peaks in scramble-KD MEF cells following 30 min of TNF-α stimulation versus magnitude of induction relative to unstimulated cells. Dotted line represents a log_2_FC cutoff of 1. (Right) Venn diagram representing distribution of ChIP-seq peaks at various genomic loci. (**C**) Profile plot showing RelA ChIP-Seq read density over all TNF-α induced peaks in KD cell lines. A window of ±1000 bp around the peak center is displayed. Peak positions for different KD cell lines are marked in the plot using dashed lines. (**D**) Motif enrichment analysis identified the κB sequence consensus as the top motif. Listed below are the consensus motifs for relevant cofactors. The Nfatc1, Nfat5, and Tead3 motifs overlap partially with the NF-κB consensus, whereas Cux1 and Smad4 motifs do not. (**E**) Significantly enriched terms from gene ontology analysis of biological process pathways of genes with RelA-induced peaks.

To examine the influence of these TF-CoFs in genome-wide binding pattern of RelA, we performed ChIP followed by sequencing (ChIP-seq) of TNF-α stimulated (0- and 30-min) KD MEFs with a RelA-specific antibody (GEO series GSE287359, Supplemental Table S6). Analysis of the duplicate (biological) datasets identified 9096 TNF-α-inducible (at 30-min) RelA peaks (*P*-value < 1E−5) in these samples (Fig. 4B, left). Consistent with earlier reports, the majority of the RelA binding sites in the scramble-KD control cells were observed in the intergenic regions (40.9%) or gene bodies, with only a small percentage (∼11%) in the promoter region (defined as spanning −1000 to +100 bp region of the TSS) (Fig. 4B, right). Reduced binding of RelA was observed at several sites in the KD MEF cell lines, although enhancement was also observed at a few sites. Overall, the analysis of changes in RelA binding at the TNF-α-induced peaks revealed greatest perturbation by KD of Nfat5 and Cux1, followed by that of Nfatc1 and Smad4 (Fig. 4C and Supplementary Fig. S4C). Depletion of Nfatc1 and Nfat5 revealed a common change in pattern of RelA recruitment, suggesting their similar binding or competitive modalities. KD of Tead3 affected binding of RelA to only a few promoters and rather enhanced recruitment of RelA at various sites, suggesting a possible role of Tead3 as a competitive repressor of RelA. When RelA ChIP-seq signal is normalized over all identified peaks, Tead3 depletion indicated a minimal effect, and Cux1 depletion had a maximal effect, with the rest in between (Fig. 4C). Overall, recruitment of RelA binding was affected at a significant number of sites in at least one of the co-TF KDs, with recruitment at a few promoters being perturbed by all five KDs. Genome browser tracks revealed significant reduction of specific peaks at known NF-κB target genes—*Rgs19 (in* Nfatc1-KD), *Ccl8 (in* Nfat5-KD), and *Cd44* (in Cux1-KD) and *Cxcl2* (in Nfat5-, Cux1-, and Smad4-KD) cells (Supplementary Fig. S4D). Furthermore, we observed that binding of Cxcl2 promoter DNA is significantly lower to NF-κB from TNF-α-stimulated nuclear extracts of Nfatc1-KD cells than NF-κB from control nuclear extract, suggesting that differences in promoter binding can be captured *in vitro* (Supplementary Fig. S4D). Further ChIP-based assessment in WT cells could provide a clearer understanding of the correlation of binding of Nfatc1 or Cux1 to promoters of genes that showed reduced RelA binding.

We further investigated the possibility of these DNA-binding TFs influencing recruitment of RelA through binding their own DNA REs that are either overlapping with the κB site(s) or present nearby. Motif analysis revealed that consensus κB sites were the primary TF binding sites (log *P*-value = −2.8E4) in control MEF cells (Fig. 4D) and these were associated with the genes involved in the inflammatory pathways (Fig. 4E). Tead3, Nfatc1, and Nfat5 consensus sequences were enriched at cross-linked sites; however, those of Cux1 and Smad4 were not (Fig. 4D). Since NF-κB binding sites display significant overlap with Tead3, Nfatc1, and Nfat5, it is challenging to ascertain whether these influence RelA recruitment by binding directly to κB sites. Furthermore, Cux1 and Smad4 appear to function in RelA recruitment through protein–protein interactions, likely, if not exclusively, by direct RelA interaction. Overall, these results suggest that certain cofactors could support recruitment of RelA to selective promoters through their direct interaction with RelA, although TF-cofactors with overlapping sites could certainly compete and affect recruitment of RelA at other promoters.

### TF-cofactors regulate gene expression by RelA

**Figure 5.**
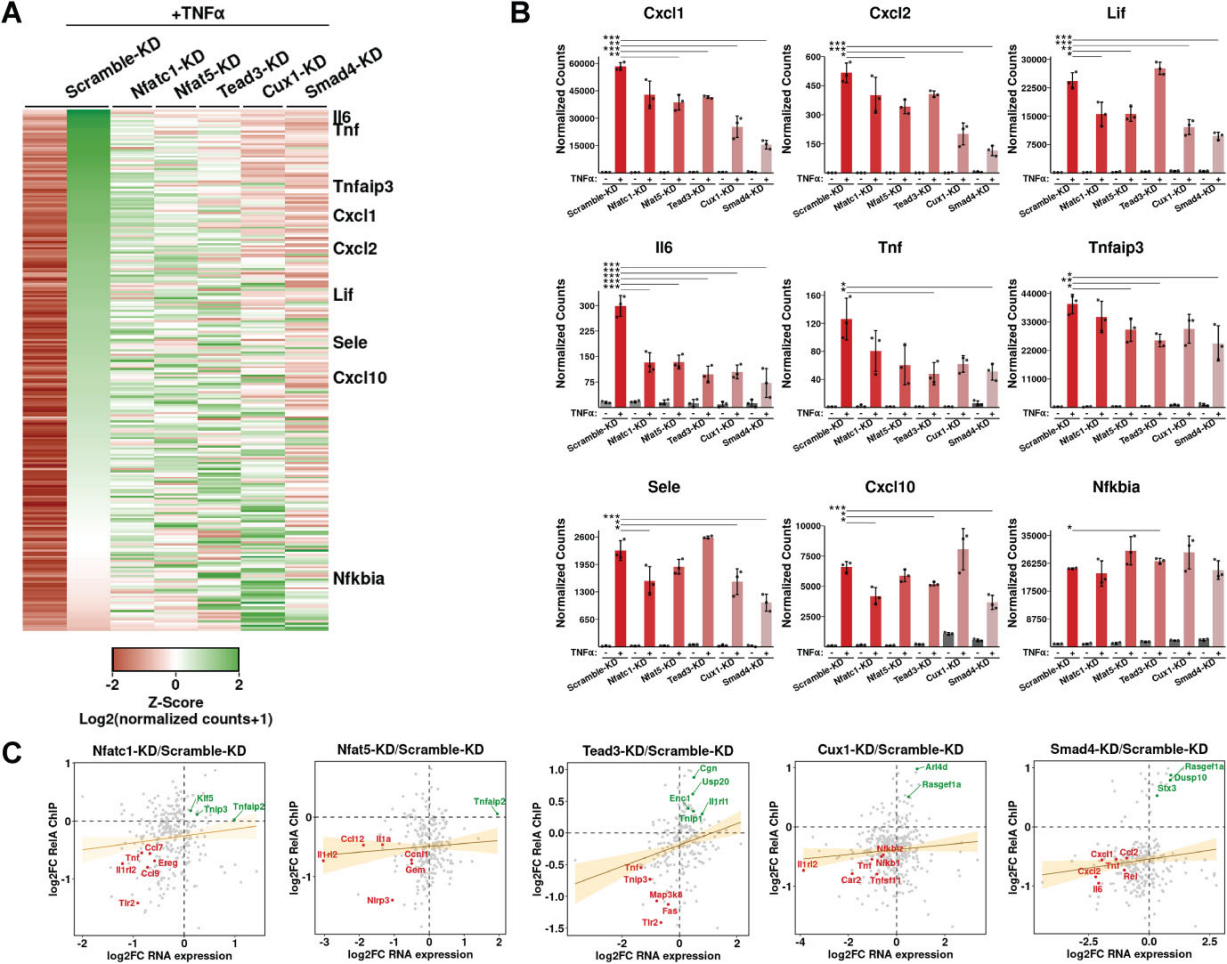
Response of TNF-α on expression of genes in various KD cell lines by RNA-seq-based transcriptomic analysis. (**A**) Heatmap representation of normalized *Z*-scores of transcript count for TNF-α induced genes in KD cell lines (log_2_FC > 0.5, *P*-value < .01, *n* = 250 genes in uninduced versus TNF-α treated Scramble-KD cells). Cells were stimulated with TNF-α for 1 h before RNA isolation. Genes are sorted based on highest to lowest *Z*-scores of differential expressions in Scrambled-KD sample. Data represented are average of *n* = 3 replicates for each condition. (**B**) Normalized counts of nine well-characterized RelA target genes in different KD cells presented as bar plots. Error bar represents SD. *n* = 3 independent experimental replicates. *P < .05, **P < .01, ***P < .005. *P*-value was calculated by Wald test. (**C**) Plot of ChIP-seq to RNA-seq correlation in TNF-α-treated KD MEF cells compared to Scramble-KD control cells. The plots display log_2_FC of normalized transcript counts against log_2_FC in ChIP-Seq binding intensity (*P*-value < .05) for TNF-α induced genes (log_2_FC > 0, *P*-value < .05 in control cells) for CoF-KD relative to Scramble-KD control cells. Plots represent average of triplicate RNA-seq and duplicate RelA ChIP-seq data. A linear regression line is shown and shaded area in orange indicates 95% confidence interval.

To test whether a reduced level of above-mentioned TF-cofactors alters recruitment of RelA to promoters, we performed genome-wide bulk RNA sequencing of the control and five KD MEF cells (used in the ChIP experiments) post TNF-α induction of 1 h (GEO series GSE287360, Supplementary Table S7). Analysis of triplicate (biological) datasets revealed that TNF-α induction increased expression of 250 genes in control scramble-KD cells (log_2_FC > 1, *P*-value < .01), with many linked to immune response and cytokine activity pathways, as expected. Further analysis revealed genes that were differentially expressed in the TNF-α-induced KD cells when compared to TNF-α-induced control cells (*P*-value < .05; Fig. 5A). These genes were arranged in order of highest to lowest transcript levels measured in TNF-α induced control (scramble-KD) cells and displayed along with the transcript levels observed in the five KD cells using a heatmap (Fig. 5A). The expression patterns differ among the KD cell lines, in contrast to the observance of rather similar RelA ChIP signals in Nfat family KD cells. Altered expression of genes was most pronounced in Smad4-KD cells (128/250 genes, *P*-value < .05) despite the apparent minor role of Smad4 compared to the other cofactors in recruitment of RelA. Each KD affected a distinct set of genes, with some commonality among them. The differential expression of canonical NF-κB target genes is presented in Fig. 5B. Expression of IL6 was affected in all five KD cells, whereas that of Nfkbia was not affected in any. The expression levels of most cytokine genes were impacted by KD of one or more of these TF-CoFs. These results allude to a distinct regulatory makeup of individual RelA-regulated promoters.

The changes in transcript levels do not appear to be correlated with changes in ChIP-seq scores in KD cells (Fig. 5C and Supplementary Fig. S5A); however, the extent of a ChIP score reduction in KD cells did not match with the extent of transcript reduction. For example, in Nfatc1-KD cells, we observe that a higher percentage of genes with reduced ChIP-seq scores also had reduced transcript levels (160/210 genes with reduced RelA binding). In this KD, ChIP-seq scores were also enhanced for several genes (83/293) of which 18 displayed a higher expression. It is possible that the observed difference in effect among genes, i.e. increased or decreased ChIP-seq scores or transcript level, may be stemming from multi-modal effects of these TF-CoFs—in some cases binding to RelA and helping its recruitment, and in some cases their direct engagement to overlapping or nearby promoter elements, thereby antagonizing binding of RelA. Higher transcript levels of many genes with corresponding higher RelA ChIP scores were observed in Tead3 KD cells supporting a role of Tead3 in acting as a repressor of RelA (57/74 genes with increased RelA binding) (Fig. 5C). We analyzed whether the affected genes in KD cells are part of specific biological functions and observed no noticeable associations. Since no distinct correlation in changes in RelA ChIP and transcript levels among KD and control cells are observed, this could imply that RNA levels are being influenced by other post-transcriptional processes [30]. Overall, these results represent a rather complex relationship of TF-CoFs and recruitment of RelA.

### Clustered κB sites in the promoter/enhancer region of Cxcl1 facilitate transcription along with TF-cofactors

We explored the possible combinatorial role of clustered weak κB sites and TF-CoFs in supporting recruitment of NF-κB to promoter using the Cxcl1 gene as a model system. Analysis of 500-bp region around the transcription start site (TSS) of Cxcl1 gene (Fig. 1A) revealed a 150-bp segment containing at least five κB sites, both strong and weak. The strongest κB site is located 57 bp upstream of TSS. RelA ChIP-seq also revealed another peak within a putative enhancer region located 15 kb upstream of the TSS containing a strong (GGGATTTCCC; *Z*-score 8.0) and four weak κB sites within a ∼150 bp segment (Supplementary Fig. S6A) [30]. Binding of RelA to both the enhancer and the promoter regions was reduced in Nfatc1-KD, Nfat5-KD, Cux1-KD, and Smad4-KD cells (Supplementary Fig. S6B).

**Figure 6.**
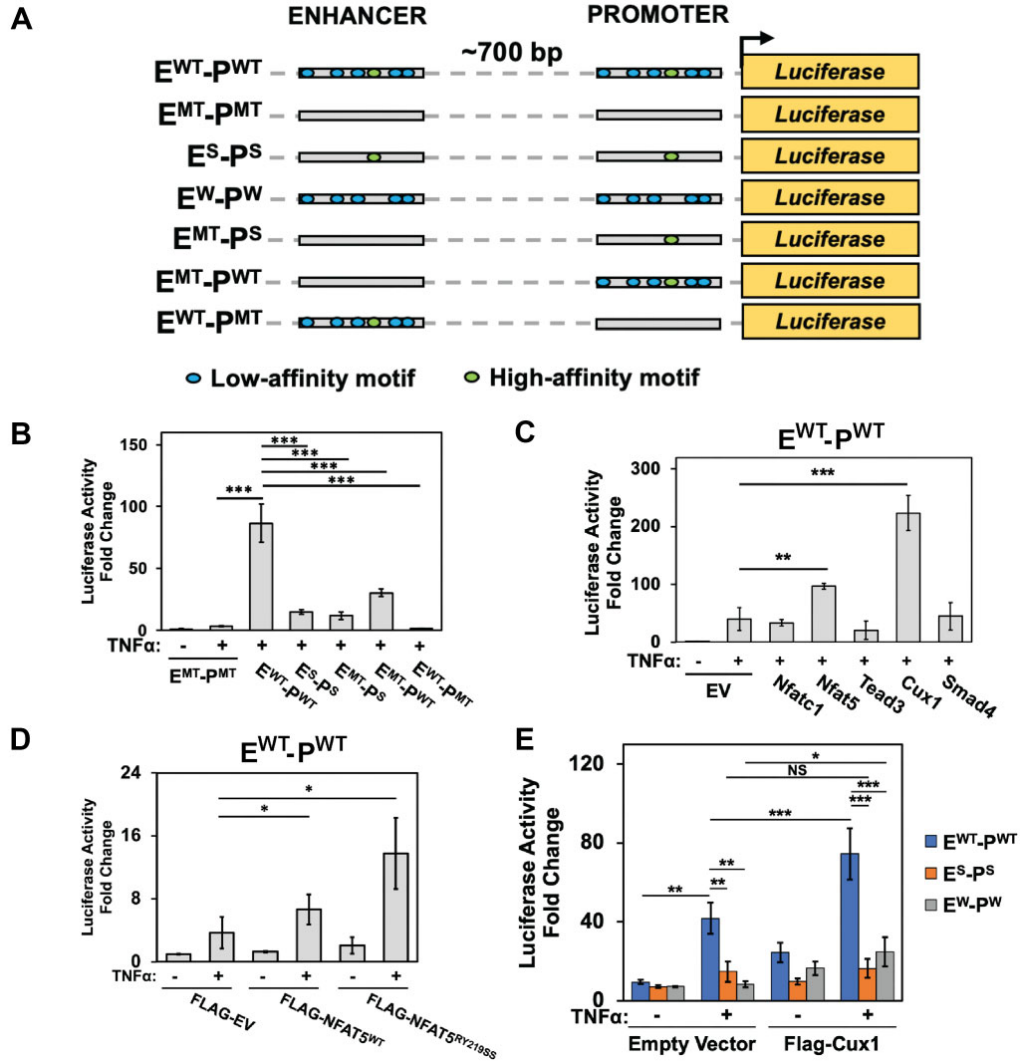
Clustered κB sites at the Cxcl1 enhancer and promoter regions regulate RelA-mediated transcriptional activation. (**A**) A schematic of WT and mutant luciferase reporter constructs under Cxcl1 regulatory regions (enhancer and promoter) containing multiple κB sites. The core enhancer and promoter regions are separated by a 700 bp fragment situated upstream of the promoter and downstream of the enhancer. Green and blue circles represent high- and low-affinity κB motifs. (**B**) Plot of luciferase reporter activity (see “Materials and methods” section) from various Cxcl1 enhancer–promoter hybrid constructs assayed in HeLa cells. (**C, D**) Plot of luciferase activity from Cxcl1 E^WT^-P^WT^ luciferase construct cotransfected with control EV or Flag-tagged cofactors in HeLa cells. Fold changes are relative to unstimulated E^MT^-P^MT^ activity. (**E**) Plot of Cux1-dependent luciferase activity from Cxcl1 luciferase constructs containing both strong and weak sites (blue), strong sites only (orange), and weak sites only (gray) in HeLa cells. Fold changes are relative to unstimulated E^MT^-P^MT^ activity.

We assessed functionality of these κB sites within the promoter and enhancer regions of the Cxcl1 gene, using luciferase reporter assay. The reporter constructs were under control of WT or mutant (MT) promoter (P)–enhancer (E) hybrid elements (Fig. 6A and Supplementary Table S4), where the promoter and enhancer are separated by a 700 bp spacer adapted from an upstream region of Cxcl1 promoter. The WT construct (E^WT^-P^WT^) exhibited ∼20–30-fold enhanced reporter activity compared to the mutant construct (E^MT^-P^MT^) with all κB sites mutated (Fig. 6B). The expression with construct containing mutated enhancer κB sites (E^MT^-P^WT^) was reduced to ∼30%, whereas that with construct containing mutated promoter κB sites (E^WT^-P^MT^) was reduced to ∼10%. The expression construct driven by strong sites of both the promoter and enhancer elements (E^S^-P^S^) led to a 3.5-fold increase, i.e. ∼10% of the activity from E^WT^-P^WT^ construct, whereas that driven by just the strongest κB site of the promoter (E^MT^-P^S^) displayed only a two-fold increase in luciferase activity. These results strongly suggest (i) a cooperation between the enhancer and promoter elements and (ii) a potent role of weak κB sites in boosting functionality of strong sites present in both the promoter and enhancer regions (Fig. 6B).

Next, we assessed expression potential of these reporters under conditions where the putative TF-cofactors were overexpressed. Overexpression of Nfat5 caused a significant enhancement in reporter expression, which was much more enhanced upon overexpression of Cux1; however, overexpression of Nfatc1, Smad4, and Tead3 displayed no discernible effect (Fig. 6C). Previously, we observed a reduction of RelA ChIP-seq scores in all TF-cofactor KD cells except for Tead3. It is possible that use of truncated Cux1 and Nfatc1 (Cux1^762–1516^ and Nfatc1^1–698^) rendered these as activators or repressors, respectively. To further assess whether TF-cofactors could be functioning without requiring their DNA binding activity, we compared reporter activity from E^WT^-P^WT^ construct upon overexpression of either WT Nfat5 or its DNA-contacting residues mutated mutant (RY219SS). A slight enhancement in reporter activity was observed with mutant Nfat5 compared to WT (Fig. 6D). We verified that the corresponding mutation in recombinant Nfatc1 (R439E) mutant does not bind DNA by EMSA, whereas neither the recombinant WT nor mutant Nfat5 RHR displayed observable binding by EMSA, perhaps due to distinct binding mechanisms (Nfatc1 binds DNA primarily as a monomer and Nfat5 as a dimer) (Supplementary Fig. S6C–E). We also examined the effect of Cux1 on reporter activity from both weak (E^W^-P^W^) and strong (E^S^-P^S^) constructs. Although both mutants displayed a marked reduction in reporter expression, the presence of Cux1 significantly activated reporter expression from the E^W^-P^W^ construct containing only the weak sites. Its effect on the E^S^-P^S^ was not statistically significant (Fig. 6E). None of these weak sites bear resemblances to Cux1 consensus sequence. These results support the possibility of a TF-cofactor, such as Cux1, likely enhancing recruitment of RelA using its non-DNA-binding activity and acting primarily through weak κB sites. Collectively, these data hint at a collaboration between κB sites and a large set of TF-cofactors in recruiting NF-κB to enhancer/promoter regions.

## Discussion

In this study, we observed that promoters of genes rapidly activated by RelA dimers in response to stimuli often contain not one but multiple κB sites present in clusters. We explored the role of these κB sites of a broad spectrum of binding affinity in recruitment of NF-κB RelA dimers to promoter and in transcription. Most κB sites within the cluster exhibited rather a weak affinity *in vitro* toward RelA dimers. In parallel, we also observed association of many accessory factors, including some other DNA-binding TFs, to promoter elements bound to NF-κB RelA. Analysis of these DNA-binding TFs indicates that they regulate recruitment of RelA to the transcription site using both their DNA-binding and non-DNA-binding activities.

### Clustering of weak and strong κB sites

We analyzed the presence of multiple κB sites within the promoter and enhancer regions of several rapid response murine NF-κB target genes—primarily cytokines (e.g. Cxcl1, Cxcl2, and Tnfa) and NF-κB/IκB family members (e.g. Nfbia, Nfkid, Nfkb1, and Nfkb2). The κB sites appeared to be clustered within segments of 150–200 bp, a length that corresponds to nucleosome-free DNA regulatory regions in activated genes [31]. These rapid response promoters usually contained one strong site with multiple weak sites clustered around it. The *in vitro* affinity of individual weak κB sites to NF-κB RelA homodimer or p50:RelA heterodimer is often undetectable (i.e. Kd > ∼1500 nM). These sites often deviated from the consensus at several bases. Interestingly, X-ray structural studies revealed that the global nature of protein–DNA interaction is preserved regardless of the difference in binding affinity and despite both the protein and DNA undergoing conformational adjustments in forming the complex.

### Significance of weak κB sites

The functional significance of weak TF-binding sites has been recognized, particularly in regulation of developmental genes of lower eukaryotes [18, 21, 22]. However, their mechanistic role(s) in facilitating formation of transcription complexes remain unclear. We explored this significance by mutating weak sites in vicinity of the putative strong site in promoter of five rapid response RelA target genes involved in the non-developmental programs. In all cases, we observed diminished levels of RelA recruitment and transcription, thereby underscoring a functional role of weak sites in the enhancer–promoter–TSS region. Reporter assays further demonstrated synergistic effects of artificially clustered κB sites in activating transcription. Since an *in vitro* study indicated a lack of cooperativity through tandem κB sites in recruiting RelA dimers [33], we anticipate alternative mechanisms underlying multi-site cooperativity possibly functioning not through DNA [32].

### Role of other TFs in recruiting RelA

We observed association of various factors, including multiple TF-cofactors, at the clustered κB site region of a native promoter and tested whether they regulate recruitment of NF-κB RelA dimers. Characterization of five of these TF-cofactors indicated their interaction with RelA to be weak. However, a subset of these TF-cofactors collaborated with the κB sites in their native context to help recruit RelA at specific transcription sites. It is possible that a cofactor tags multiple RelA dimers using their different domains and facilitates recruitment of RelA to a κB cluster. Alternatively, multiple interacting cofactors can establish a protein network at a specific subset of κB sites in the enhancer/promoter of a target gene and guide RelA to that region.

Some TF-cofactors (e.g. Cux1 and Smad4) appear to function without requiring their own DNA binding since no enrichment of their binding sites was observed at RelA-bound genomic regions. However, it is possible that these cofactors could regulate RelA recruitment through contacting DNA. This is particularly true when DNA recognition sites of TF-CoFs overlap with κB sites. To test whether DNA-binding of Nfat5 influences recruitment of RelA, we measured transcriptional potential of a DNA-binding defective mutant of Nfat5 in a reporter assay. The mutant showed an enhancement albeit minor of the reporter expression, suggesting a possibility of these TFs to regulate recruitment without involving their DNA-binding activity. This observation agrees with reports indicating recruitment of TFs to genomic regions lacking their cognate DNA sites [34]. In contrast, the removal of Tead3 enhanced gene expression, consistent with its competition with RelA for strong κB sites binding. This is expected since its recognition site is closest to κB sites among the five TF-cofactors.

### Role of enzymes and RBPs in recruiting RelA

We postulate that activated nuclear RelA is trapped by various cofactors at promoter region, thus effectively enhancing concentration of RelA and enabling its recruitment to weak sites. Cofactors not belonging to TF-cofactors perhaps facilitate recruitment of RelA by different means, such as by modifying RelA. The association of kinases, methylases, acetylases, deacetylases, and glycosylases at RelA target promoters prompts us to propose that these enzymes could be modifying both protein and DNA and regulating assembly or disassembly of transcription complex at the start site [35–39]. The RNA-binding proteins may simultaneously engage with RNAs, promoter DNA, and RelA to stabilize the transcription complex [40–43].

### κB site clusters in recruiting RelA

The requirement of κB sites within a cluster for recruiting RelA is unclear. Our findings demonstrate that cooperativity through protein–protein interactions between two NF-κB dimers bound to tandem sites is not a general mechanism for RelA recruitment. Although such cooperative mechanisms have been demonstrated for some TFs, particularly those that bind weakly as a monomer and require a supporting factor for stable binding, this does not appear to be the case for RelA [44]. Although we categorize weak κB sites as a single class, their sequences and possibly their affinities for RelA vary substantially. For instance, deletion of a single weak κB site among three in the Nfkbid promoter led to only a partial reduction in both RelA binding and transcriptional activity. If all κB sites were equally important and acted simultaneously and cooperatively, we would have observed more pronounced defects. As shown in Fig. 1A, the promoter and possible enhancers of rapidly and strongly activating NF-κB genes have more than three weak κB sites. At least in case of the Cxcl1 promoter-enhancer, the collective activity of the ∼10 weak κB sites (the precise number of sites that can engage RelA is not experimentally assessed) is comparable to that of two strong κB sites. Transcriptional activation from the weak κB sites appears to require Cux1, unlike that from the strong sites, supporting a role of Cux1 in recruitment of RelA to promoters with weak sites. Importantly, cofactor engagement appears to be stimulus specific (different cofactors are activated by TNFα and LPS) and temporally regulated, suggesting a model of dynamic cooperativity for transcriptional synergy. This dynamic and flexible regulatory mechanism offers a broader range of control than a rigid cooperative model. It also implies that single bp changes can modulate activity, contributing to creating a malleable complex finely tuned by both protein and DNA modifications [27, 45].

### Multi-site and multi-factor-based recruitment of a TF to enhancer-coupled promoter

We observed a strong contribution of weak TF-binding sites present in the enhancer and the promoter regions through reporter expression assays. This revealed intriguing possibilities of a multisite collaboration in facile recruitment of a TF. We speculate that clustered sites within both the enhancer and promoter regions capture multiple copies of the target TF in association with multiple other TFs. Central to this model is the idea that the TF triggering activation is stabilized at the promoter with help of a dynamic condensate that includes juxtaposed promoter and enhancer regions enabled through chromatin looping and a large body of multiple factors. This enhancer–promoter communication provides much greater combinatorial possibilities of protein–protein interactions for a finer regulatory control compared to a TF binding to tandem sites in a linear promoter region. This is particularly significant in context of promoters and enhancers enriched with weak TF binding sites, which cannot stably recruit transcriptional machinery on their own but provide critical support for TF binding to strong sites. A cartoon highlighting proposed formation of essential bridges through weak interactions among multiple sites and multiple factors supporting DNA-bound TF RelA for a robust and timely transcriptional output is presented in the graphical abstract. We surmise that this principle is likely part of other stimulus-induced transcription programs, where dynamic enhancer–promoter interactions, cofactor activities, and coordinated TF binding together shape the transcriptional response with high specificity and flexibility. It should be noted that alternative mechanisms of TF recruitment are also possible where the promoter contains one or more strong binding sites of a single TF or multiple TFs, allowing efficient transcription with minimal to no need for enhancers or cofactors. This may be particularly relevant in sustained high-level expression of housekeeping genes.

Overall, this study points to a significant complexity of RelA-targeted promoter elements that cannot be explained by NF-κB RelA binding directly to a high-affinity cognate κB DNA. We observe cooperativity among multiple NF-κB dimers *in vitro* with κB sites that are spaced apart and not tandem. Our findings also allude to engagement of not only NF-κB RelA but also multiple other factors, including other DNA-binding cofactors—hinting at a multi-protein transcription assembly involving multiple κB sites that likely allows capture of NF-κB at the promoter even at a low concentration and activates transcription through mediator complexes and RNA polymerase II.

## Supplementary Material

gkaf846_Supplemental_Files

## Data Availability

Data used in this study are made available through Supplementary data file and public repositories. Mass spectrometry data can be accessed at PRoteomics IDEntifications Database (PRIDE) under the accession PXD065004. ChIP-seq and RNA-seq data can be accessed at https://www.ncbi.nlm.nih.gov/geo/query/acc.cgi?acc=GSE287359 and at https://www.ncbi.nlm.nih.gov/geo/query/acc.cgi?acc=GSE287360, respectively. X-ray data can be accessed at https://www.rcsb.org/structure/9E6W.
