# Supplementary material for: Teamwork of clustered low-affinity κB sites and accessory factors regulates transcriptional strength of NF-κB RelA dimers
Source: Nucleic Acids Res. 2025 Oct 8;53(18):gkaf846. doi: 10.1093/nar/gkaf846 (PMC12507517; doi:10.1093/nar/gkaf846)
Supplement: gkaf846_Supplemental_Files [file gkaf846_supplemental_files.zip › Supplementary_Information_NAR_2ndRevision.pdf]

Supplementary Information for

Teamwork of clustered low-affinity κB sites and accessory factors regulates transcriptional strength of NF-κB RelA dimers

Shandy Shahabi[1], Tapan Biswas[1], Yuting Shen[1], Rose Sanahmadi[1], Yaya Zou[1], and Gourisankar Ghosh[1,5]

Corresponding Author: Dr. Gourisankar Ghosh
E-mail: gghosh@ucsd.edu

This PDF file includes:

Supplementary Figures S1a-d, S2a-b, S3a-f, S4a-e, S5, S6a-e and their legends
Supplementary Table S1 (Table attached as a separate .xlsx file)
Supplementary Table S2 (Table attached as a separate .csv file, legend included here)
Supplementary Table S3 (Table and legend)
Supplementary Table S4 (Table attached as a separate .csv file, legend included here)
Supplementary Table S5 (Table attached as a separate .xlsx file, legend included here)
Supplementary Table S6 GEO Series records for ChIP-seq Data
Supplementary Table S7 GEO Series records for RNA-seq Data

Supplementary Figures

Supplementary Figure 1.

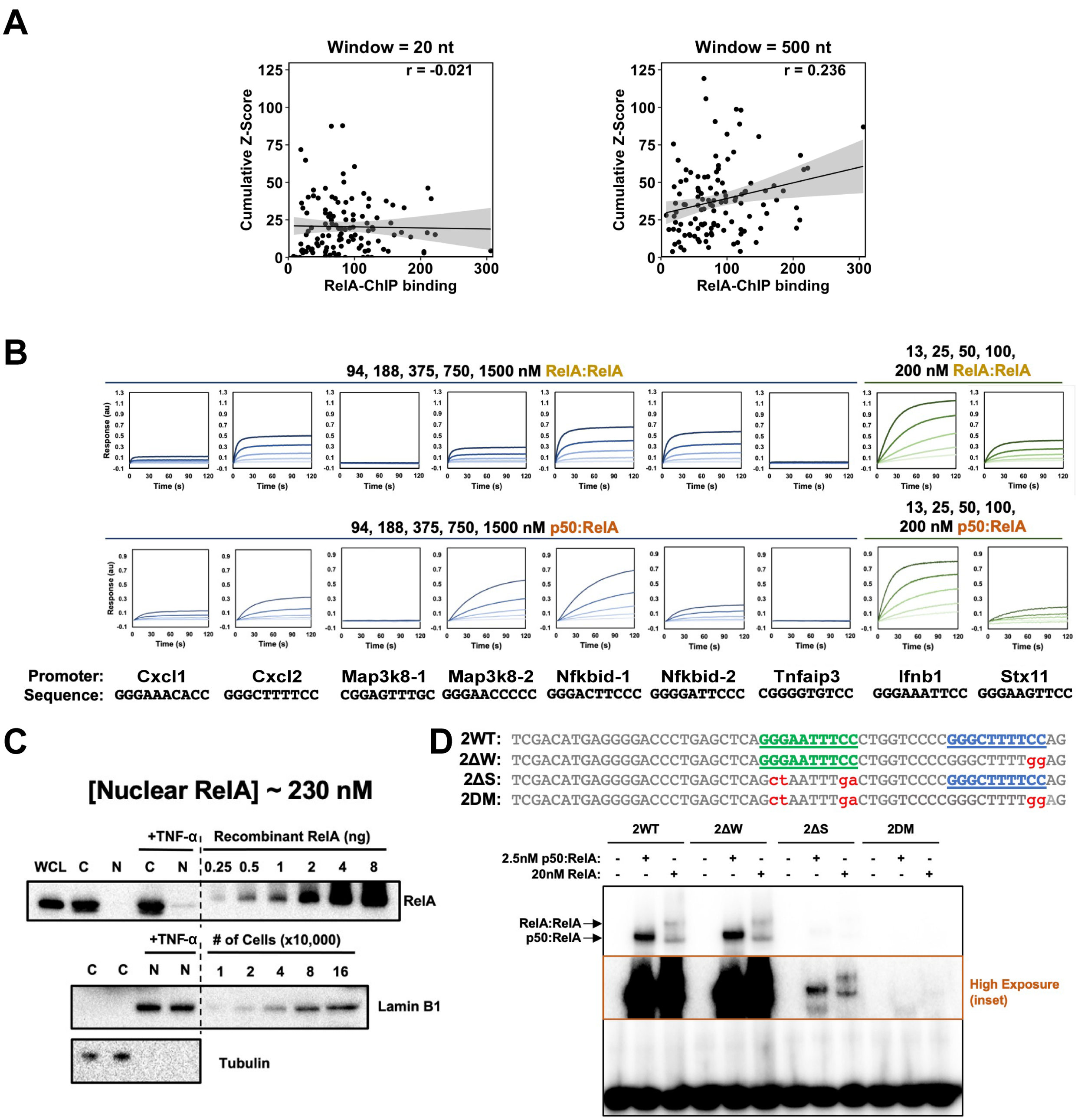

Supplementary Figure 1.

A. Pearson's correlation plots between RelA ChIP-seq score and cumulative Z-score with a 20-bp window (left) or a 500-bp window (right) around the strong site. A linear regression line is shown in black and shaded area indicates 95% confidence. n = 116 peaks analyzed.

B. The binding of κB site sequences to full-length RelA homodimer (top) and p50:RelA heterodimer (bottom) by BioLayer interferometry assay. Binding to Ifnb1 and Stx11 κB sites, present in many promoters, are used as references of affinity. Concentrations of RelA homodimer or p50:RelA heterodimer used in the assay are indicated above plots.

C. Immunoblot analysis of RelA from whole cell lysate (WCL), cytoplasm (C), and nuclear (N) fractions of HeLa S3 cells with or without TNF-α stimulation. Purified recombinant RelA was used as a standard for quantitation of RelA amount, and Lamin B1 was used as a standard for estimating cell number.

D. EMSA of wild-type and mutant promoter sequences derived from Cxcl2 gene with RelA homodimer and p50:RelA heterodimer. Strong and weak κB site sequences are colored in green and blue, respectively, and mutated nucleotides are denoted in red. A high exposure inset is marked with a brown border.

Supplementary Figure 2

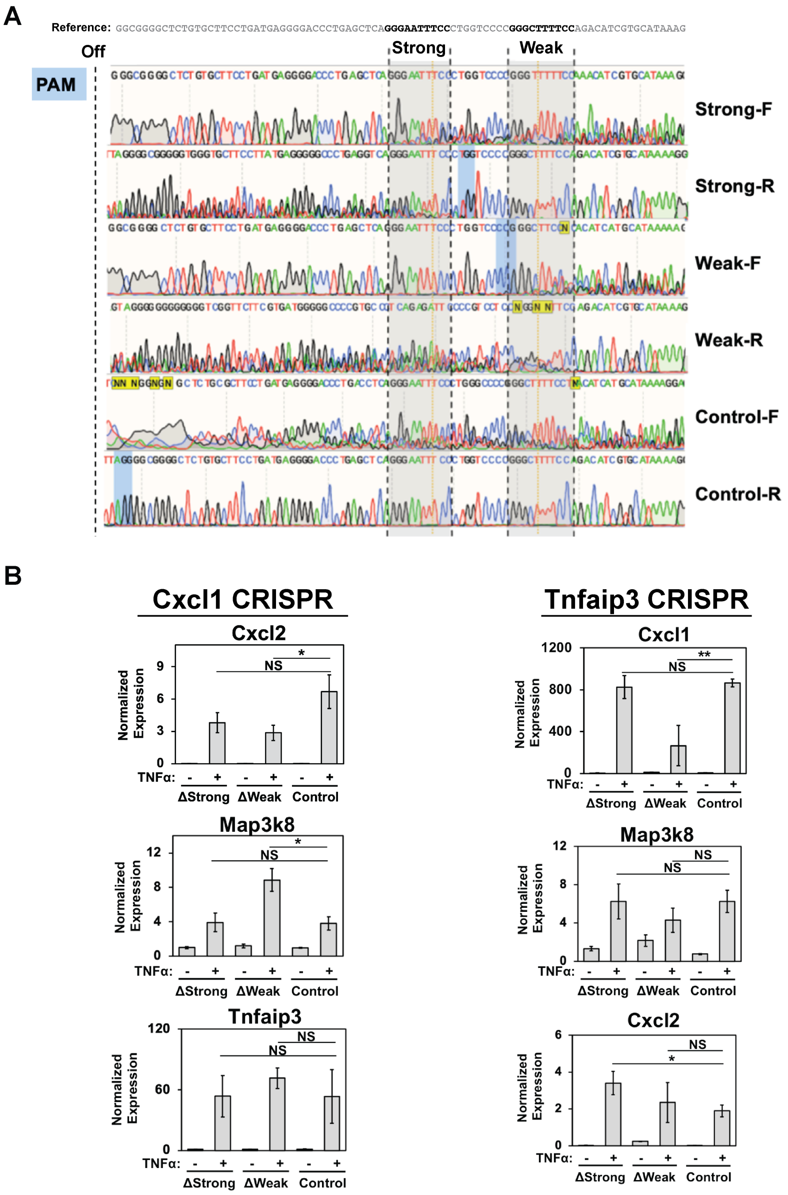

Supplementary Figure 2.

A. Sanger sequencing for verification of CRISPR-based mutagenesis of Cxcl2 promoter in MEF cells. Bulk cell sequencing was performed on a 200 bp amplicon of the Cxcl2 promoter region generated by PCR of gDNA from corresponding CRISPR-modified cell lines. Traces represent Sanger sequencing results in both the forward and reverse direction, with perturbations to sequencing traces indicating upstream genomic modifications. The mm10 reference sequence of the Cxcl2 promoter is shown above. The PAM sequences used to generate the three mutants are highlighted in blue shades.

B. Normalized transcript levels of corresponding off-target genes upon CRISPR-mediated targeting of strong, weak, or control sites of Cxcl1 (left) or Tnfaip3 (right) measured by RT-qPCR. MEF cells were stimulated with TNF-α for 1 h. Data represented are normalized by Gapdh expression. *$P < 0.05$, ** $P < 0.001$, NS = non-significant. P value was calculated by one-tailed Student's t-test. For all data points, n = 3 experimental replicates. Error bar represent standard deviation.

Supplementary Figure 3

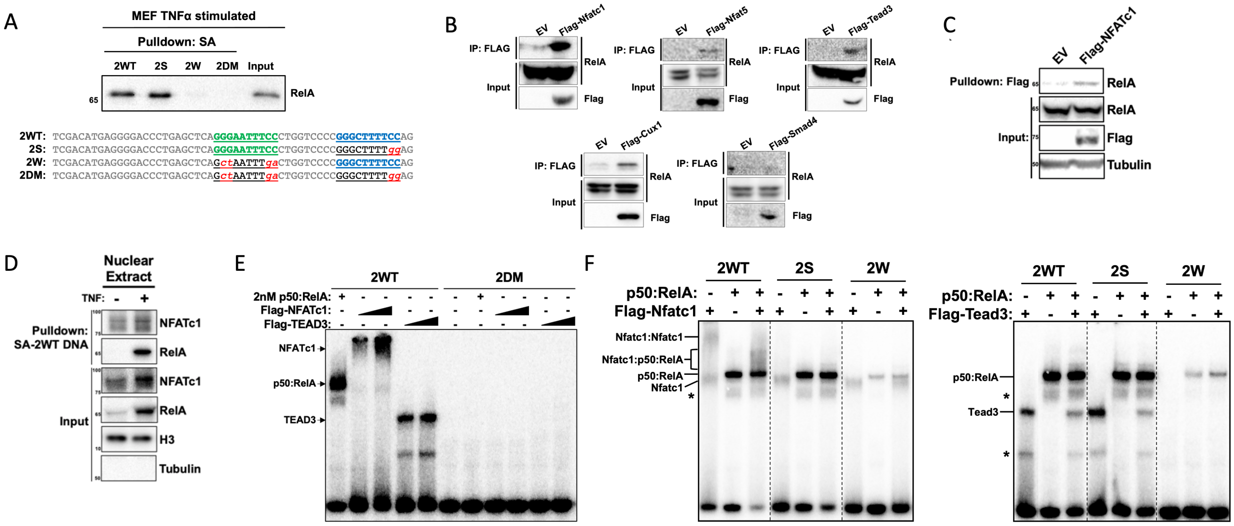

Supplementary Figure 3.

A. Immunoblot analysis of pulldowns with biotinylated wild-type and mutant Cxcl2 DNA from TNF-α-induced (30 min) MEF nuclear extracts using RelA-specific antibody. The sequences DNA are indicated below. The high affinity κB-site highlighted in green, the low affinity κB-motif in blue, and mutations in red.

B. Immunoblots from Flag pulldown assay with extracts of TNF-α-induced HEK293T co-transfected with Flag-tagged Nfatc1, Nfat5, Tead3, Cux1, or Smad4 and HA-tagged RelA. Transfection of empty vector was used as a control.

C. Immunoblot analysis of endogenous RelA immunoprecipitated using anti-Flag beads from cell lysate of HEK293T cells transfected with Flag-tagged Nfatc1 or empty control vector.

D. Pulldown assay using biotinylated wild-type Cxcl2 promoter DNA-streptavidin bead with nuclear extract of MEF cells without or with TNF-α stimulation.

E. Native polyacrylamide EMSA with radiolabeled WT or double mutant Cxcl2 promoter DNA probes and purified recombinant p50:RelA heterodimer, Flag-NFATc1, or Flag-TEAD3. DNA sequences are listed in **A.**

F. Native polyacrylamide EMSA with radiolabeled WT or mutant Cxcl2 promoter DNA (listed below) and recombinant p50:RelA and Flag-Nfatc1 (left) or Flag-Tead3 (right) * nonspecific protein band. DNA sequences are listed in **A**.

Supplementary Figure 4

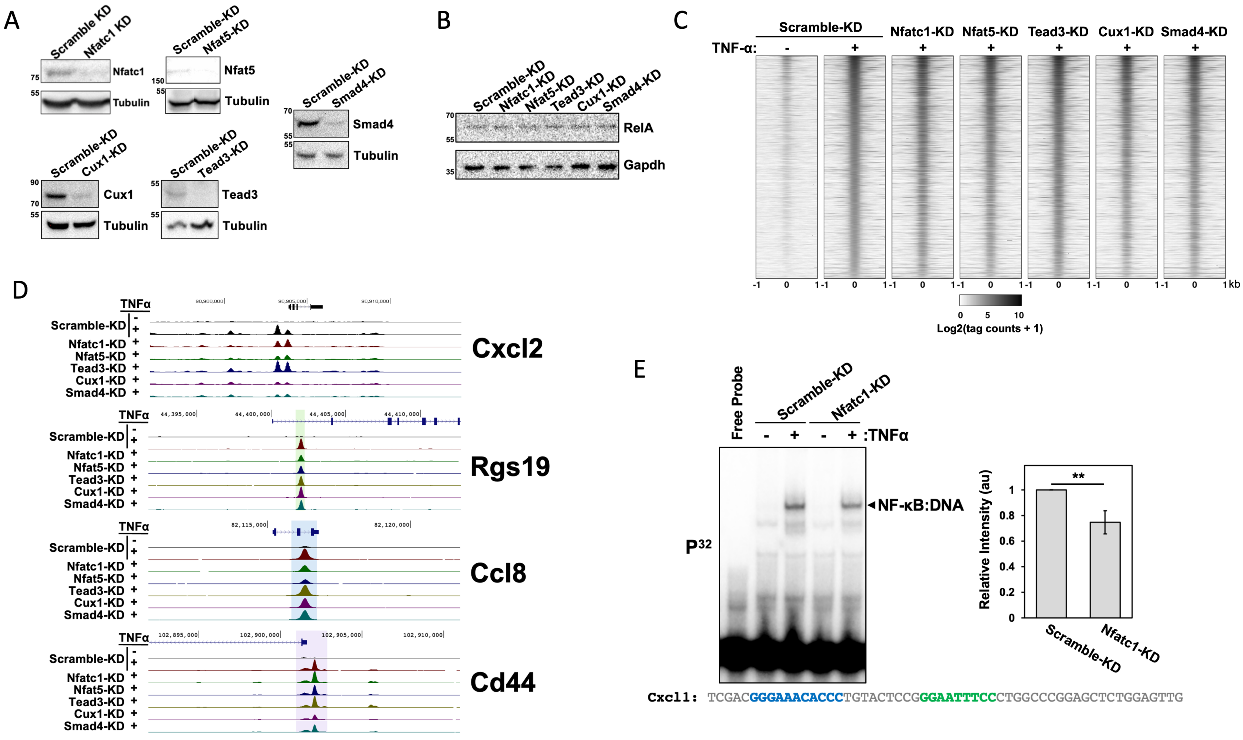

Supplemental Figure 4. RelA ChIP of five cofactors and a scramble (control) KD MEF cell lines.
A. Western blot of whole cell lysate from KD cell lines showing efficiency of KD. Level of tubulin is used to normalize loading.
B. Western blot of whole cell lysate showing relative levels of RelA protein across all KD cell lines. Level of Gapdh is used to normalize loading.
C. Histogram representation of log transformed RelA ChIP-Seq tag counts for RelA-induced peaks (log2FC > 1, p-value < 0.05, n = 14581 peaks in uninduced to TNF-α treated Scramble-KD cells) across all TNF-α stimulated KD cell lines. A window of +/- 1000 bp around the peak center is displayed. Peaks are sorted in decreasing order of total intensity observed in TNF-α stimulated control Scramble-KD cells.
D. Genome browser tracks of RelA induced ChIP-Seq peaks in all KD cell lines showing differential effects of knockdowns at the target genes Cxcl2, Rgs19, Ccl8, and Cd44. RelA ChIP-Seq peak areas with differential reduction are highlighted in green (Nfat5-KD), blue (Nfatc1-KD and Nfat5-KD), and purple (Cux1-KD).
E. (left) Native polyacrylamide EMSA with radiolabeled Cxcl1 promoter DNA (listed below) using nuclear extract of TNF-α stimulated (30 min) Scramble-KD and Nfatc1-KD MEF cell lines. (right) Quantification of relative NF-κB binding to Cxcl1 DNA with extracts of Scramble-KD and Nfac1-KD cell lines using a Bar Plot. **P < 0.01. P value was calculated by one-tailed Student's t-test. n = 3 independent experimental replicates. Error bar represents standard deviation.

Supplementary Figure 5

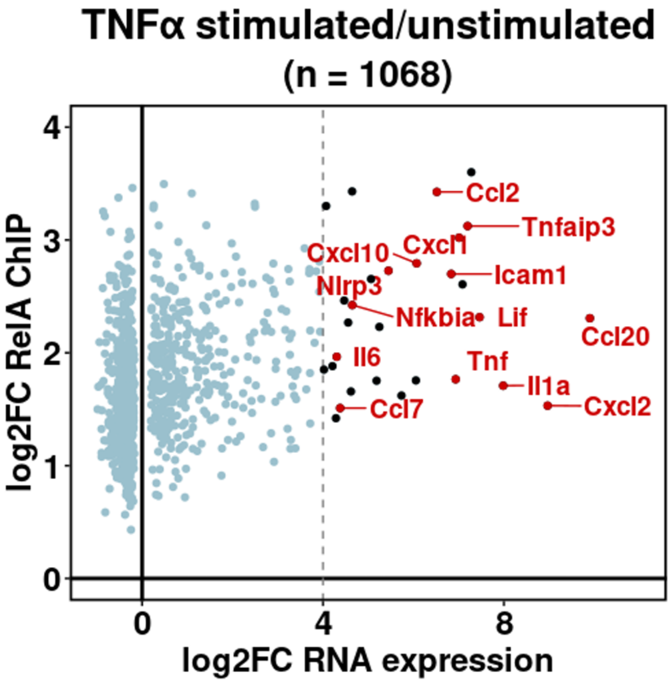

Supplementary Figure 5.

Correlation between changes in RelA ChIP-Seq signal and RNA expression in Scramble-KD cells. Genes were filtered to include significantly differentially expressed genes (p value < 0.05, Wald test) with induced RelA peaks (p value < 1E-5, Wald). Known NF-κB targets are labeled in red. Fold change calculations are based on triplicate RNA-Seq and duplicate ChIP-Seq datasets. n = 1068 data points analyzed.

Supplementary Figure 6

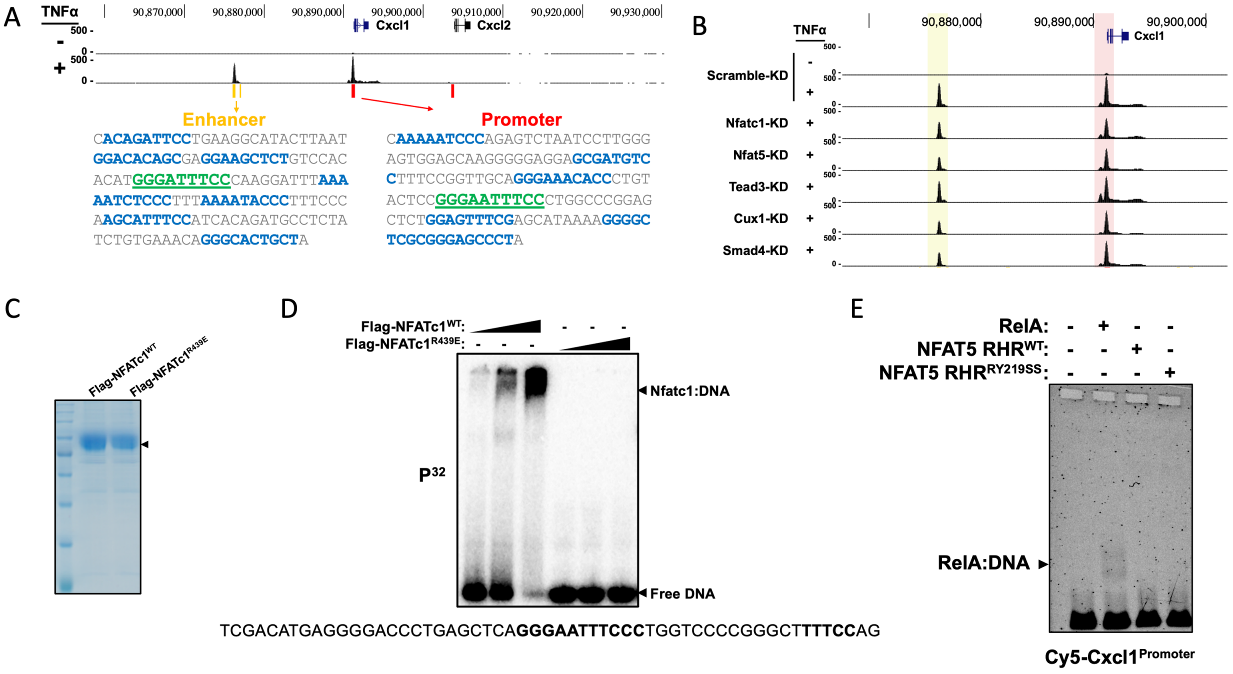

Supplementary Figure 6.

A. Genome browser representation of RelA ChIP-Seq signal at the Cxcl1-Cxcl2 locus in TNF-α stimulated (30 min) control cells. Yellow and red lines below tracks indicate predicted enhancer and promoter regions in the ENCODE database. The mm10 genomic sequences of the enhancer and promoter regions are also shown, with the central high-affinity κB motif highlighted in green and putative low-affinity κB sites in blue. The enhancer and promoter regions are separated by approximately 15 kbp.

B. Genome browser tracks of RelA induced ChIP-Seq peaks at the Cxcl1 enhancer and promoter regions of all KD MEF cell lines. Red and yellow shades highlight RelA binding regions at the promoter and enhancer.

C. Purified Flag-Nfatc1^{WT} and Flag-Nfatc1^{R439E} from transfected HEK 293T cells resolved in a 10% SDS-polyacrylamide gel and Coomassie stained. Cells were transfected for 24 h prior to harvesting for purification. The leftmost lane is of the molecular weight marker, indicating size of the purified proteins to be approximately 78 kDa.

D. Native polyacrylamide EMSA of radiolabeled Cxcl1 promoter DNA (listed below) with increasing concentrations of purified Flag-Nfatc1, either WT or DNA-binding deficient mutant. The DNA sequence is displayed below with binding motifs for Nfatc1 in bold letters.

E. Native agarose EMSA of Cy5-labeled Cxcl1 promoter DNA with purified recombinant His-tagged RelA, Nfat5 RHR, or DNA binding deficient mutant Nfat5 RHR^{RY219SS}.

Supplementary Table S1. Sequences of DNA and DNA primers

(Table attached as a separate file denoted as SupplementaryTable1.xlsx)

Supplementary Table S2.

Bioinformatic analysis of RelA-binding genomic regions. The 500-bp sequence of genomic regions identified from ChIP-seq data, along with the ChIP-seq scores; Also included are the putative κB sites of this segment identified from a PBM study along with the individual Z-scores.

(Table attached as a separate file denoted as SupplementaryTable2.csv)

Supplementary Table S3

	**Structural Model**		RelA:κB DNA (Cxcl2 weak T-centric)
	**Deposition ID**		9E6W
	**Data Collection**		
	Wavelength (Å)		0.97946
	Resolution range (Å)		50.0-2.03(2.07-2.03)
	Space group		P 21 21 2
	**Cell dimensions**		
	a, b, c (Å)		118.79 133.08 45.36
	α, β, γ (°)		90.000 90.000 90.000
	Total reflections		604462
	Unique reflections		46623
	Redundancy		13.0(10.6)
	Completeness (%)		99.4(99.5)
	I/σ(I)		94.9/3.1(2.7/2.1)
	Rpim		0.022(0.547)
	CC1/2 /(CC*)		1.00(0.681)/1.00(0.90)
	**Refinement**		
	Resolution range (Å)		37.48-2.04(2.10-2.04)
	No. of reflections (work/test set)		35763/1752
	Rwork/Rfree		0.244/0.283
	Number of non-hydrogen atoms		5386
	**r.m.s. deviations**		
	Bonds ()		0.0025
	Angles (*)		0.9609
	**Ramachandran plot**		
	Favored (%)/allowed(%)		96/4
	Outliers (%)		0
	Rotamer outliers (%)		2
	Clashscore		7
	Average B-factor (Å^2)		35

Supplementary Table S3: Crystallographic data used in obtaining the structural model of RelA:κB DNA (Cxcl2 weak) complex, and refinement statistics of the model.

Supplementary Table S4.

Luciferase reporter expression assay data. The experimental values from individual experiments of the luciferase reporter expression assays (Figures 2D, E and 6A-E), along with the calculation of fold-increase in expression values for each set.

(Table attached as a separate .csv file denoted as SupplementaryTable4.csv)

Supplementary Table S5.

Total counts of identified proteins in MEF nuclear extract pulled down by biotinylated κB-DNA:RelA using mass-spectrometry. Individual results of experimental duplicates for each type of DNA used in pulldown and duration of TNF-α treatment are provided.

(Table attached as a separate file denoted as SupplementaryTable5.xlsx)

Supplementary Table S6. GEO Series records for ChIP-seq Data: GSE287359

GSM8744856	Scramble-KD	MEF unstim	ChIP-seq	(replicate	1)	Jan	22,	2025
GSM8744857	Scramble-KD	MEF unstim	ChIP-seq	(replicate	2)	Jan	22,	2025
GSM8744858	Scramble-KD	MEF TNF	ChIP-seq	(replicate	1)	Jan	22,	2025
GSM8744859	Scramble-KD	MEF TNF	ChIP-seq	(replicate	2)	Jan	22,	2025
GSM8744860	Nfatc1-KD	MEF TNF	ChIP-seq	(replicate	1)	Jan	22,	2025
GSM8744861	Nfatc1-KD	MEF TNF	ChIP-seq	(replicate	2)	Jan	22,	2025
GSM8744862	Nfat5-KD	MEF TNF	ChIP-seq	(replicate	1)	Jan	22,	2025
GSM8744863	Nfat5-KD	MEF TNF	ChIP-seq	(replicate	2)	Jan	22,	2025
GSM8744864	Tead3-KD	MEF TNF	ChIP-seq	(replicate	1)	Jan	22,	2025
GSM8744865	Tead3-KD	MEF TNF	ChIP-seq	(replicate	2)	Jan	22,	2025
GSM8744866	Cux1-KD	MEF TNF	ChIP-seq	(replicate	1)	Jan	22,	2025
GSM8744867	Cux1-KD	MEF TNF	ChIP-seq	(replicate	2)	Jan	22,	2025
GSM8744868	Smad4-KD	MEF TNF	ChIP-seq	(replicate	1)	Jan	22,	2025
GSM8744869	Smad4-KD	MEF TNF	ChIP-seq	(replicate	2)	Jan	22,	2025

Supplementary Table S7. GEO Series records for RNA-seq Data: GSE287360

GSM8744870	Scramble-KD	MEF	unstim	RNA-seq	(replicate	1)	Jan	22,	2025
GSM8744871	Scramble-KD	MEF	unstim	RNA-seq	(replicate	2)	Jan	22,	2025
GSM8744872	Scramble-KD	MEF	unstim	RNA-seq	(replicate	3)	Jan	22,	2025
GSM8744873	Scramble-KD	MEF	TNF	RNA-seq	(replicate	1)	Jan	22,	2025
GSM8744874	Scramble-KD	MEF	TNF	RNA-seq	(replicate	2)	Jan	22,	2025
GSM8744875	Scramble-KD	MEF	TNF	RNA-seq	(replicate	3)	Jan	22,	2025
GSM8744876	Nfatc1-KD	MEF	unstim	RNA-seq	(replicate	1)	Jan	22,	2025
GSM8744877	Nfatc1-KD	MEF	unstim	RNA-seq	(replicate	2)	Jan	22,	2025
GSM8744878	Nfatc1-KD	MEF	unstim	RNA-seq	(replicate	3)	Jan	22,	2025
GSM8744879	Nfatc1-KD	MEF	TNF	RNA-seq	(replicate	1)	Jan	22,	2025
GSM8744880	Nfatc1-KD	MEF	TNF	RNA-seq	(replicate	2)	Jan	22,	2025
GSM8744881	Nfatc1-KD	MEF	TNF	RNA-seq	(replicate	3)	Jan	22,	2025
GSM8744882	Nfat5-KD	MEF	unstim	RNA-seq	(replicate	1)	Jan	22,	2025
GSM8744883	Nfat5-KD	MEF	unstim	RNA-seq	(replicate	2)	Jan	22,	2025
GSM8744884	Nfat5-KD	MEF	unstim	RNA-seq	(replicate	3)	Jan	22,	2025
GSM8744885	Nfat5-KD	MEF	TNF	RNA-seq	(replicate	1)	Jan	22,	2025
GSM8744886	Nfat5-KD	MEF	TNF	RNA-seq	(replicate	2)	Jan	22,	2025
GSM8744887	Nfat5-KD	MEF	TNF	RNA-seq	(replicate	3)	Jan	22,	2025
GSM8744888	Tead3-KD	MEF	unstim	RNA-seq	(replicate	1)	Jan	22,	2025
GSM8744889	Tead3-KD	MEF	unstim	RNA-seq	(replicate	2)	Jan	22,	2025
GSM8744890	Tead3-KD	MEF	unstim	RNA-seq	(replicate	3)	Jan	22,	2025
GSM8744891	Tead3-KD	MEF	TNF	RNA-seq	(replicate	1)	Jan	22,	2025
GSM8744892	Tead3-KD	MEF	TNF	RNA-seq	(replicate	2)	Jan	22,	2025
GSM8744893	Tead3-KD	MEF	TNF	RNA-seq	(replicate	3)	Jan	22,	2025
GSM8744894	Cux1-KD	MEF	unstim	RNA-seq	(replicate	1)	Jan	22,	2025
GSM8744895	Cux1-KD	MEF	unstim	RNA-seq	(replicate	2)	Jan	22,	2025
GSM8744896	Cux1-KD	MEF	unstim	RNA-seq	(replicate	3)	Jan	22,	2025
GSM8744897	Cux1-KD	MEF	TNF	RNA-seq	(replicate	1)	Jan	22,	2025
GSM8744898	Cux1-KD	MEF	TNF	RNA-seq	(replicate	2)	Jan	22,	2025
GSM8744899	Cux1-KD	MEF	TNF	RNA-seq	(replicate	3)	Jan	22,	2025
GSM8744900	Smad4-KD	MEF	unstim	RNA-seq	(replicate	1)	Jan	22,	2025
GSM8744901	Smad4-KD	MEF	unstim	RNA-seq	(replicate	2)	Jan	22,	2025
GSM8744902	Smad4-KD	MEF	unstim	RNA-seq	(replicate	3)	Jan	22,	2025
GSM8744903	Smad4-KD	MEF	TNF	RNA-seq	(replicate	1)	Jan	22,	2025
GSM8744904	Smad4-KD	MEF	TNF	RNA-seq	(replicate	2)	Jan	22,	2025
GSM8744905	Smad4-KD	MEF	TNF	RNA-seq	(replicate	3)	Jan	22,	2025
